# *FUS*-ALS hiPSC-derived astrocytes impair human motor units through both gain-of-toxicity and loss-of-support mechanisms

**DOI:** 10.1186/s13024-022-00591-3

**Published:** 2023-01-18

**Authors:** Katarina Stoklund Dittlau, Lisanne Terrie, Pieter Baatsen, Axelle Kerstens, Lim De Swert, Rekin’s Janky, Nikky Corthout, Pegah Masrori, Philip Van Damme, Poul Hyttel, Morten Meyer, Lieven Thorrez, Kristine Freude, Ludo Van Den Bosch

**Affiliations:** 1 Department of Neurosciences, Experimental Neurology and Leuven Brain Institute, KU Leuven – University of Leuven, 3000 Leuven, Belgium; 2 VIB Center for Brain & Disease Research, Laboratory of Neurobiology, 3000 Leuven, Belgium; 3 Department of Development and Regeneration, KU Leuven – University of Leuven, Campus Kulak, 8500 Kortrijk, Belgium; 4 KU Leuven – University of Leuven, EM-Platform of the VIB Bio Imaging Core and VIB Center for Brain and Disease Research, Research Group Molecular Neurobiology, 3000 Leuven, Belgium; 5 KU Leuven – University of Leuven, VIB Bio Imaging Core; VIB Center for Brain & Disease Research, 3000 Leuven, Belgium; 6 VIB Nucleomics Core, VIB, Herestraat 49, 3000 Leuven, Belgium; 7 Department of Neurology, University Hospitals Leuven, 3000 Leuven, Belgium; 8 Department of Veterinary and Animal Sciences, Faculty of Health and Medical Sciences, University of Copenhagen, 1870 Frederiksberg C, Denmark; 9 Department of Neurology, Odense University Hospital, 5000 Odense, Denmark; 10 Department of Neurobiology Research, Institute of Molecular Medicine, University of Southern Denmark, 5000 Odense, Denmark

**Keywords:** Amyotrophic lateral sclerosis, Astrocyte, Reactivity, Cytokines, Motor unit, Microfluidic, Neuromuscular junction, WNT/β-catenin pathway

## Abstract

**Background:**

Astrocytes play a crucial, yet not fully elucidated role in the selective motor neuron pathology in amyotrophic lateral sclerosis (ALS). Among other responsibilities, astrocytes provide important neuronal homeostatic support, however this function is highly compromised in ALS. The establishment of fully human coculture systems can be used to further study the underlying mechanisms of the dysfunctional intercellular interplay, and has the potential to provide a platform for revealing novel therapeutic entry points.

**Methods:**

In this study, we characterised human induced pluripotent stem cell (hiPSC)-derived astrocytes from *FUS*-ALS patients, and incorporated these cells into a human motor unit microfluidics model to investigate the astrocytic effect on hiPSC-derived motor neuron network and functional neuromuscular junctions (NMJs) using immunocytochemistry and live-cell recordings. *FUS*-ALS cocultures were systematically compared to their CRISPR-Cas9 gene-edited isogenic control systems.

**Results:**

We observed a dysregulation of astrocyte homeostasis, which resulted in a *FUS*-ALS-mediated increase in reactivity and secretion of inflammatory cytokines. Upon coculture with motor neurons and myotubes, we detected a cytotoxic effect on motor neuron-neurite outgrowth, NMJ formation and functionality, which was improved or fully rescued by isogenic control astrocytes. We demonstrate that ALS astrocytes have both a gain-of-toxicity and loss-of-support function involving the WNT/β-catenin pathway, ultimately contributing to the disruption of motor neuron homeostasis, intercellular networks and NMJs.

**Conclusions:**

Our findings shine light on a complex, yet highly important role of astrocytes in ALS, and provides further insight in to their pathological mechanisms.

**Graphical Abstract:**

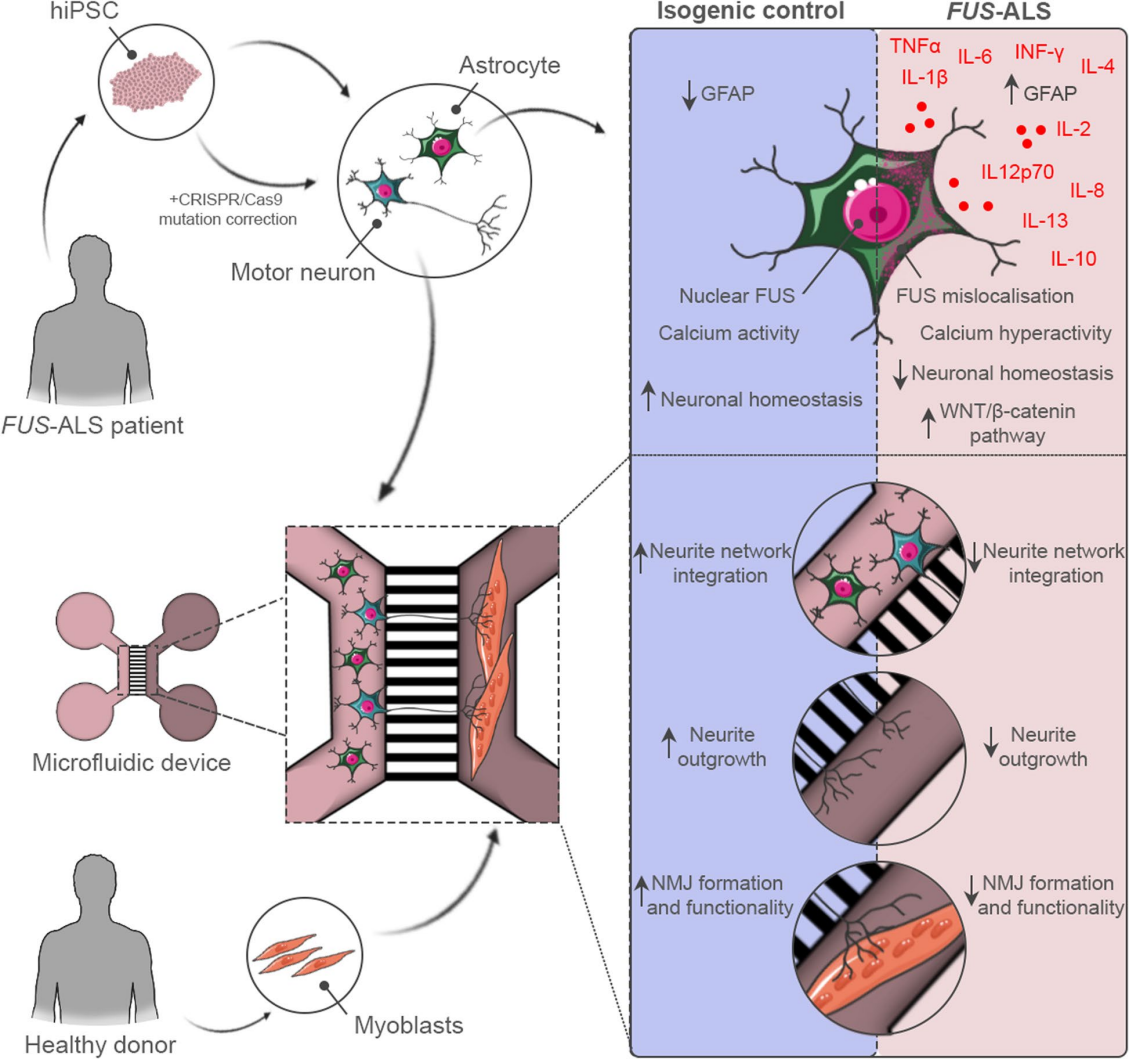

**Supplementary Information:**

The online version contains supplementary material available at 10.1186/s13024-022-00591-3.

## Background

Amyotrophic lateral sclerosis (ALS) is a progressive motor neuron disorder in which the upper motor neurons in the motor cortex as well as the lower motor neurons in the brainstem and the ventral horn of the spinal cord undergo selective and progressive cell death. Symptoms include muscle weakness, spasticity, hyperreflexia, fasciculations, and muscle atrophy. The rapid progression of the disease limits median survival after symptom onset to 2 to 5 years mostly due to respiratory failure [1], and currently no effective treatment is available. In 90% of cases, ALS is a sporadic disease (sALS) clinically indistinguishable from inherited familial forms, but with an unknown aetiology [2]. The remaining 10% covers familial ALS (fALS) cases where mutations in the *FUS RNA binding protein* (*FUS*) gene are the fourth most prevalent cause of fALS in the Western world (2.8%), and the second most common form in the Asian ALS population (6.4%) [3]. In addition, *FUS* mutations cause the majority of juvenile and paediatric cases of ALS [4]. Interestingly, nucleus-to-cytoplasmic mislocalisation of FUS protein, which is a hallmark of *FUS* mutant pathology, has also been documented in sALS cases [5], and FUS-positive inclusions are present in approximately 10% of frontotemporal lobar degeneration (FTLD) cases despite the lack of genetic mutations in the *FUS* gene [6]. This suggests that *FUS*-mediated pathology might have a more general function in neurodegenerative disorders.

Despite the selective degeneration of motor neurons, several studies demonstrate an important role for glial cells in ALS [7–10]. Astrocyte reactivity is a prominent hallmark in ALS patients [7, 11, 12], and the number of reactive astrocytes has been shown to correlate with disease progression [13]. Normally, astrocytes are the key players in maintaining homeostasis and optimal neuronal function through synaptic activity and plasticity modulation, inter-neuronal crosstalk, nutrition distribution, waste removal and structural support [14]. However, studies in ALS show that astrocytes promote motor neuron toxicity through reduced glutamate uptake [15–17], reduced lactate production and shuttling [18, 19] and increased secretion of toxic factors such as tumour necrosis factor α (TNF-α) and reactive oxygen species [20–24]. Despite this insight, many aspects of the astrocytes’ pathologic mechanism in ALS are not fully known.

In this study, we aimed to unravel the role of hiPSC-derived astrocytes in the context of *FUS*-ALS and include these cells in our previously established human motor unit microfluidics model [25, 26] to evaluate the astrocytic effect in a novel multicellular system. We discovered that *FUS*-ALS astrocytes showed increased reactivity and secretion of inflammatory cytokines. Once incorporated into the motor unit system, the mutant astrocytes impaired the neurite outgrowth as well as NMJ formation and functionality. Our data establish a mutant *FUS*-mediated dysregulation of astrocytes, which causes a synergistic gain-of-toxicity and loss-of-support functionality. We propose an auto-regulatory role, in which astrocytes unsuccessfully attempt to counteract this toxicity through secretion of anti-inflammatory cytokines and upregulation of the WNT/β-catenin pathway in motor neurons.

## Methods

### Cell lines and reagents

Two heterozygous *FUS*-mutant hiPSC lines from a 17-year-old male ALS patient with a de novo mutation (P525L) and a 71-year-old female ALS patient (R521H) were each compared to their corresponding CRISPR-Cas9 gene-edited isogenic control hiPSC line (P525P and R521R) [25–28]. The isogenic control hiPSC lines were made by CellSystems (Troisdorf, Germany). hiPSC lines were cultured using Complete Essential 8™ medium (Cat N° A1517001) with 1% penicillin/streptomycin (Cat N° 15070063) on Geltrex®- (Cat N° A1413301) coated plates. Human myoblasts were harvested from biopsies obtained from a healthy 60-year-old male donor via the Human Body Donation program of KU Leuven (KULAK). All cells were routinely tested for mycoplasma contamination with MycoAlert Mycoplasma Detection Kit (Lonza, Rockland, ME, USA, Cat N° LT07-318). Chemicals and reagents used for cell culture were purchased from Thermo Fisher Scientific (Waltham, MA, USA) unless otherwise specified (see Supplementary Table 1, Additional file 1).

### Generation of hiPSC-derived astrocytes

hiPSC-derived astrocytes were generated using a slightly modified version of a recently published protocol [29]. In brief, hiPSCs were dissociated with collagenase type IV (Cat N° 10780004), and the cell clusters were collected in Corning® ultra-low attachment flasks (Sigma-Aldrich, St. Louis, MO, USA, Cat N° 734–4140) to support embryoid body (EB) formation. EBs were kept in neuronal induction medium (NIM) consisting of Complete Essential 8™ medium with 1% penicillin/streptomycin supplemented with 0.1 μM LDN-193189 (Stemgent, Beltsville, MA, USA, Cat N° 04–0074-02) and 10 μM SB431542 (Tocris Bioscience, Bristol, UK, Cat N° 1614) for the first week of differentiation. Media changes were performed on days 1, 2 and 4. On day 7, the EBs were plated on Geltrex®-coated plates for neural rosette formation and subsequent neural progenitor cell (NPC) expansion in neuronal maturation medium (NMM) containing 50% DMEM/F12 (Cat N° 11330032) and 50% Neurobasal medium (Cat N° 21103049) with 1% L-glutamine (Cat N° 25030–024), 1% penicillin/streptomycin, 1% N-2 supplement (Cat N° 17502–048) and 2% B-27™ without vitamin A (Cat N° 12587–010) supplemented with 10 ng/ml recombinant murine fibroblast growth factor (FGF)-2 (PeproTech, Rocky Hill, NJ, USA, Cat N° 450–33), 10 ng/ml recombinant human epidermal growth factor (EGF) (ProSpec, Rotherham, UK, Cat N° CYT-217), 0.1 μM LDN-193189 and 10 μM SB431542. The medium was changed daily and NPCs were passaged a few times using accutase (Sigma-Aldrich, Cat N° A6964). On day 16, NPCs were cultured until day 25 in astrocyte differentiation medium (ADM) made of 90% Neurobasal medium, 1% penicillin/streptomycin, 1% N-2 supplement, 1% non-essential amino acids (Cat N° 11140050) and 0.8 μM ascorbic acid (Sigma-Aldrich, Cat N° A4403) supplemented with 10 ng/ml FGF-2, 200 ng/ml recombinant human insulin-like growth factor (IGF)-1 (Peprotech, Cat N° 100–11), 10 ng/ml human Activin A (Cat N° PHC9564) and 10 ng/ml recombinant human Heregulinβ1 (Peprotech, Cat N° 100–03) to convert NPCs to astrocyte progenitor cells (APCs). Medium changes were made every second day during the period. On day 25 (d25/d+0), a glial switch is expected to occur, which commences the astrocyte maturation period. APCs were plated for final experiments or for expansion on new Geltrex®-coated plates and matured for an additional 4 weeks in astrocyte maturation medium (AMM) consisting of 50% DMEM/F12, 50% Neurobasal medium, 1% non-essential amino acids, 1% N-2 supplement, 1% L-glutamine, 1% penicillin/streptomycin, 2% fetal bovine serum (FBS) (Cat N° 10270106), 0.8 μM ascorbic acid, and 1% sodium pyruvate (Cat N° 11360–070) supplemented with 200 ng/ml IGF-1, 10 ng/ml Activin A and 10 ng/ml Heregulinβ1. The medium was changed every second day and maturing astrocytes were cryopreserved in FBS and 10% DMSO (Sigma-Aldrich, Cat N° D2650-100ML) at 2 weeks of maturation for later use. For WB (western blot), qPCR, RNA-sequencing (RNAseq) and secretome experiments, hiPSC-derived APCs were plated at d25/d+0 at 50 000 cells/cm^2^ in 6-well plates (Cellstar Greiner bio-one, Kremsmünster, Austria, Cat N° 657160) and allowed to mature for 1–4 weeks without passages.

### Generation of hiPSC-derived motor neurons

hiPSC-derived motor neurons were generated using a previously published protocol [25–27], which is a modified version of the Maury et al. protocol [30]. Briefly, hiPSCs were dissociated with collagenase IV and the cell clusters were transferred to Corning® ultra-low attachment flasks to promote EB formation. EBs were cultured in neuronal medium consisting of 50% DMEM/F12, 50% Neurobasal medium, 1% penicillin/streptomycin, 0.5% L-glutamine, 1% N-2 supplement, 2% B-27™ without vitamin A, 0.5 μM ascorbic acid and 0.1% β-mercaptoethanol (Cat N° 31350010). On day 0 and 1 of differentiation, EBs received neuronal medium with 5 μM Y-27632 (Merck Millipore, Burlington, MA, USA, Cat N° 688001), 0.2 μM LDN-193189, 40 μM SB431542 and 3 μM CHIR99021 (Tocris Bioscience, Cat N° 4423). On days 2 and 4, the EB medium consisted of neuronal medium supplemented with 0.1 μM retinoic acid (Sigma-Aldrich, Cat N° R2625) and 500 nM smoothened agonist (Merck Millipore, Cat N° 566660). On day 7, the EB medium was changed to neuronal medium supplemented with 10 ng/ml brain-derived neurotrophic factor (BDNF) (PeproTech, Cat N° 450–02) and 10 ng/ml glial cell line-derived neurotrophic factor (GDNF) (PeproTech, Cat N° 450–10) in addition to the 0.1 μM retinoic acid and 500 nM smoothened agonist. On day 9, 10 μM DAPT (Tocris Bioscience, Cat N° 2634) was added to the fresh day 7 medium. On day 10, EBs were dissociated into NPCs using 0.05% trypsin (Gibco, Gaithersburg, MA, USA, Cat N° 25300054). NPCs were cryopreserved in knockout serum replacement (Cat N° 10828–028) with 10% DMSO or plated in day 9 medium with 1% RevitaCell™ (Cat N° A2644501) for further experiments. On day 11 of the differentiation, additional day 9 medium was added to the wells. On day 14, cells received neuronal medium supplemented with 10 ng/ml BDNF, 10 ng/ml GDNF and 20 μM DAPT. On day 16, the differentiating motor neurons received 10 ng/ml BDNF, GDNF and ciliary neurotrophic factor (CNTF) (PeproTech, Cat N° 450–13) in addition to 20 μM DAPT. From day 18 on, motor neurons were cultured in neuronal medium with 10 ng/ml BDNF, GDNF and CNTF. The medium was changed every other day until terminal differentiation.

### Human myoblasts and myotube differentiation

Human skeletal muscle cells were isolated from a human vastus lateralis muscle biopsy as described before [31]. Briefly, isolated tissue was cut in strips of approximately 2 mm × 10 mm using sterile forceps and scalpel after removing excess connective tissue and fat. Muscle strips were pinned under tension in a Sylgard silicone- (Dow Corning, Midland, MICH, USA, Cat N° 1060040) coated 6-well plate. Two days after pinning the strips, enzymatic digestion was performed by incubating the muscle strips at 37 °C for 1 h in DMEM, high glucose with pyruvate (Cat N° 31966021) supplemented with 0.1% collagenase, type II (Worthington Biochemical Corp, Lakewood, NJ, USA, Cat N° LS004176) and 4 mg/ml dispase II (Roche Diagnostics, Basel, Switzerland, Cat N° 4942078001). After the incubation, cells were collected by filtering through a 100 μm cell strainer (Corning, NY, USA, Cat N° 7340004) and fragments were incubated again to digest the whole tissue. Isolated myoblasts were pooled, centrifuged for 5 min at 200 × g and resuspended in skeletal muscle growth medium consisting of DMEM, high glucose with pyruvate, 10% FBS, 50 µg/ml gentamicin (Cat N° 15750037), and 1% Ultroser solution (Pall Corporation, NY, USA, Cat N° 15950–017). Cells were split at 60–70% confluence and used in experiments before reaching 12 doublings. Myoblasts were differentiated into multinucleated myotubes by changing the medium to DMEM, high glucose with pyruvate, 10 ng/mL recombinant human EGF (Peprotech, Cat N° AF-100–15), 10 µg/ml insulin (Sigma-Aldrich, Cat N° I9278), 50 µg/ml Gentamicin and 50 µg/mL bovine serum albumin (BSA) (Sigma-Aldrich, Cat N° A2153).

### Coculturing in microfluidic devices

hiPSC-derived motor neurons and astrocytes were cocultured in microfluidic devices with human primary myoblasts using a slightly modified version of a recently published protocol [26]. On day 10 of the coculture timeline, 250 000 motor neuron-NPCs were plated in a compartment on one side of the microgrooves of the device, while 40 000 myoblasts were plated in the opposite compartment. Motor neuron-NPCs were further differentiated following the standard protocol, while myoblasts were allowed to proliferate until day 14, where the differentiation into myotubes was initiated. On day 18, a volumetric and chemotactic gradient of 10 ng/ml BDNF, GDNF and CNTF in addition to 0.01 μg/ml recombinant human agrin protein (R&D Systems, Cat N° 6624-AG-050) and 20 μg/ml laminin (Sigma-Aldrich, Cat N° L2020-1MG) was implemented to promote motor neuron neurite migration through the microgrooves towards the myotube compartment. The myotube compartment received 200 μl/well motor neuron media with growth factors, while the motor neuron compartment received 100 μl/well without any supplements. The gradient was sustained at every media change until the end of the experiment. On day 21, 250 000 week 3.5 (d + 24) mature astrocytes were plated on top of the maturing motor neurons in the motor neuron compartment in motor neuron medium and the coculture was continued for another 1–2 weeks. Motor unit cultures without astrocytes were kept in parallel as controls. Preassembled microfluidic devices (Xona™ Microfluidics, Temecula, CA, USA, Cat N° XC150 (microgroove length: 150 μm)) were used for immunocytochemistry (ICC) and live-cell calcium recordings of NMJ functionality, while silicone microfluidic devices (Xona™ Microfluidics, Cat N° SND75 (microgroove length: 75 μm)) mounted on Aclar 33C sheets (Electron Microscopy Sciences, Hatfield, PA, USA, Cat N° 50425–25) were used for scanning electron microscopy (SEM).

### Bright-field imaging

Bright-field images of astrocyte differentiation were taken with a Nikon Eclipse Ts2 microscope with a DMK 33UX250 camera and NIS-Elements D 5.01.00 software.

### Immunocytochemistry (ICC)

ICC analyses of NMJ formation and neurite outgrowth were performed in XC150 microfluidic devices, while remaining stainings were imaged on 13 mm #1.5 coverslips (VWR, Monroeville, PA, USA, Cat N° 631-0150P) in 24-well plates (Greiner bio-one cellstar, Vilvoorde, Belgium, Cat N° 662160) using a previously described method [25, 26]. Briefly, cells were fixed using 4% paraformaldehyde (Cat N° 28908) in DPBS (Cat N° 14190250) for 15–20 min, permeabilized with 0.1% Triton X-100 (Sigma-Aldrich, Cat N° T8787) in DPBS for 20 min, and subsequently incubated with 5% normal donkey serum (NDS) (Sigma-Aldrich, Cat N° D9663) in 0.1% Triton X-100/DPBS for 30 min. Overnight incubation with primary antibodies (see Supplementary Table 1, Additional file 1) diluted in 2% NDS in 0.1% Triton X-100/DPBS was performed at 4 °C. The following day, cells were washed with DPBS and incubated with secondary antibodies in 2% NDS in 0.1% Triton X-100/DPBS for 1 h. Afterwards, nuclei were labelled with DAPI (NucBlue Live Cell Stain ReadyProbes reagent, Cat N° R37605) for 20 min. Coverslips were mounted and devices sealed with Fluorescence Mounting Medium (Dako, Glostrup, Denmark, Cat N° S3023), and images were acquired in a 1024 × 1024 format using an inverted Leica Sp8 DMI8 confocal microscope equipped with a HC PL APO CS2 10x/0.40, 20x/0.75 or HC PL APO CS 40x/0.85 dry objective lens, a Nikon A1R TiE confocal microscope equipped with a Plan Fluor 40X oil DIC objective lens or a Nikon C2 TiE confocal microscope equipped with a Plan Apo 40X oil DIC H objective lens. Analyses were performed utilizing ImageJ 1.52p and NIS-Elements AR 4.30.02 software.

### hiPSC differentiation and myotube formation analysis

hiPSC-derived motor neuron-NPCs were plated at 25 000 cells/cm^2^ and differentiated until day 28, while hiPSC-derived astrocytes were plated at 50 000 cells/cm^2^ two days before fixation and analysed at maturation weeks 1–4. Myoblasts were plated at 40 000 cells/cm^2^, differentiated into myotubes and analysed after 8 days. Images were acquired with a Leica Sp8 microscope equipped with a HC PL APO CS2 10x/0.40, 20x/0.75 or HC PL APO CS 40x/0.85 dry objective lens. The presence of cell-type-specific markers was analysed using ImageJ software. For astrocyte quantifications, a minimum of 150 cells per cell line were selected randomly based on positive DAPI staining at 20X magnification. A minimum of 300 cells were quantified per motor neuron cell line. The myotube fusion index was evaluated at 10X magnification from 15 random images from 3 individual differentiations. Myotube nuclei were counted with the ImageJ particle analyser plugin at a size of 100–500 μm^2^.

### FUS mislocalisation analysis

hiPSC-derived astrocytes were analysed at week 4 of maturation. 60 random z-stacks from 3 individual experiments were taken at 40X magnification with a Nikon C2 TiE confocal microscope equipped with a Plan Apo 40X oil DIC H objective lens and analysed using a custom automatic Nikon software script. Images were converted to maximum intensity projections, and the ratio of cytoplasm/nucleus of FUS intensity was quantified in Aquaporin-4 (AQP4)- and glial fibrillary acidic protein (GFAP)-positive astrocytes.

### Punctae ICC analysis

For ICC analysis with cleaved caspase 3, hiPSC-derived astrocytes were imaged at week 4 of maturation. 30 random images at 40X magnification from 3 individual experiments were taken per cell line using a Leica Sp8 microscope equipped with a HC PL APO CS 40x/0.85 dry objective lens and analysed using ImageJ 1.28u puncta analyser plugin. Fluorescent punctae areas (μm^2^) were calculated in each image and normalized to the number of nuclei.

### NMJ ICC analysis

NMJ formation was evaluated with ICC at days 28 and 35 of the coculture timeline. Every myosin heavy chain (MyHC)-positive myotube present in the microfluidic device channel was recorded in z-stacks. For very long myotubes, multiple z-stacks were acquired. The number of colocalisations between neurofilament heavy chain (NEFH)/synaptophysin (SYP) and α-bungarotoxin (Btx) was counted manually through each z-stack and normalized to the number of myotubes present in the z-stack. Additionally, the number of innervated myotubes were counted per image.

### β-catenin ICC analysis

Monocultured hiPSC-derived astrocytes were analysed at week 4 of maturation. 30 random z-stacks from 3 individual experiments were taken at 40X magnification with a Nikon A1R TiE confocal microscope equipped with a Plan Fluor 40X oil DIC objective lens and analysed using the same custom automatic Nikon software script as for FUS mislocalisation analysis. Images were converted to maximum intensity projections, and the ratio of nucleus/cytoplasm of β-catenin intensity was quantified in AQP4-positive astrocytes.

For cocultures, hiPSC-derived motor neuron-NPCs were plated at day 10 of differentiation at a density of 15 000 cells/cm^2^ and differentiated until day 21. At day 21 of motor neuron differentiation, astrocytes at week 3.5 of maturation (d + 24) were plated on top of the motor neurons at a density of 7 500 cells/cm^2^. The coculture was kept for 48 h in motor neuron medium before fixation. 30 random z-stacks at 12-bit and 40X magnification were taken from 3 individual experiments using a Leica Sp8 microscope equipped with a HC PL APO CS 40x/0.85 dry objective lens. For quantification of β-catenin accumulation, images were converted to maximum intensity projections and analysed using ImageJ 1.28u puncta analyser plugin. Fluorescent punctae areas (μm^2^) were calculated in each image, and the number of accumulations were quantified per βIII-tubulin-positive motor neuron. The cytoplasmic and nuclear content of β-catenin expression were quantified in βIII-tubulin-positive motor neurons using a custom automatic Nikon software script. Cytoplasmic and nuclear β-catenin intensities were normalized to the cytoplasmic and nuclear area (μm^2^), respectively, within each individual image.

### RNA-sequencing

hiPSC-derived astrocytes were collected for RNA-sequencing experiments at week 4 (d + 28) of maturation. RNA was isolated using RNeasy® Mini Kit (Qiagen, Hilden, Germany, Cat N° 74104) according to the manufacturer’s instructions.

#### RNA quality control

RNA concentration and purity were determined spectrophotometrically using the Nanodrop ND-8000 (Nanodrop Technologies) and RNA integrity was assessed using a Bioanalyzer 2100 (Agilent).

#### Library preparation

Per sample, an amount of 1 µg of total RNA was used as input. Using the Illumina TruSeq® Stranded mRNA Sample Prep Kit (protocol version: # 1000000040498 v00 October 2017) poly-A containing mRNA molecules were purified from the total RNA input using poly-T oligo-attached magnetic beads. In a reverse transcription reaction using random primers, RNA was converted into first-strand cDNA and subsequently converted into double-stranded cDNA in a second-strand cDNA synthesis reaction using DNA Polymerase I and RNAse H. The cDNA fragments were extended with a single 'A' base to the 3' ends of the blunt-ended cDNA fragments after which multiple indexing adapters were ligated introducing different barcodes for each sample. Finally, enrichment PCR was carried out to enrich those DNA fragments that have adapter molecules on both ends and to amplify the amount of DNA in the library.

#### Sequencing

Sequence-libraries of each sample were equimolarly pooled and sequenced on Illumina NovaSeq 6000 (S1 flowcell 100 cycles kit v1.5, 100 bp Single Read (100–8-8–0)) at the VIB Nucleomics Core (www.nucleomics.be).

#### Preprocessing

Low-quality ends and adapter sequences were trimmed off from the Illumina reads with FastX 0.0.14 and Cutadapt 1.15 [32, 33]. Subsequently, small reads (length < 35 bp), polyA-reads (more than 90% of the bases equal A), ambiguous reads (containing N), low-quality reads (more than 50% of the bases < Q25) and artifact reads (all but three bases in the read equal one base type) were filtered using FastX 0.0.14 and ShortRead 1.40.0 [34]. With Bowtie2 2.3.3.1 we identified and removed reads that align to phix_illumina [35].

#### Mapping

The preprocessed reads were aligned with STAR aligner v2.5.2b to the reference genome of *Homo sapiens* (GRCh38) [36]. Default STAR aligner parameter settings were used, except for ‘–outSAMprimaryFlag OneBestScore –twopassMode Basic –alignIntronMin 50 –alignIntronMax 500000 –outSAMtype BAM SortedByCoordinate’. Using Samtools 1.5, reads with a mapping quality smaller than 20 were removed from the alignments [37].

#### Counting

The number of reads in the alignments that overlap with gene features was counted with featureCounts 1.5.3 [38]. Following parameters were chosen: -Q 0 -s 2 -t exon -g gene_id. We removed genes for which all samples had less than 1 count-per-million. Raw counts were further corrected within samples for GC-content and between samples using full quantile normalization, as implemented in the EDASeq package from Bioconductor [39].

#### Differential gene expression

With the EdgeR 3.24.3 package of Bioconductor, a negative binomial generalized linear model (GLM) was fitted against the normalized counts [40]. We did not use the normalized counts directly but worked with offsets. Differential expression was tested for with a GLM likelihood ratio test, also implemented in the EdgeR package. The resulting *p*-values were corrected for multiple testing with Benjamini–Hochberg to control the false discovery rate [41]. Differentially expressed genes were selected based on False discovery rate (FDR) < 0.05. log_2_FC < -1.0 were considered downregulated, and log_2_FC > 1.0 were considered upregulated. *VCP, FUS* and *SOD1* mutant astrocyte transcriptomic data sets were acquired from Array Express (E-MTAB-10916) [49], while *C9orf72* mutant astrocyte transcriptomic data sets were accessed from GSE142730 [42]. The single reads FASTQ files from external experiments were processed in a similar way as the FASTQ files from this study and mapped to the same reference genome. The adapters could not be removed as we did not know the sequence. After counting, the normalization was performed for each independent experiment and control samples were discarded. As low counted genes were discarded for each experiment, only 5249 out of the 6468 initial differentially expressed genes were shared by all experiments. The R package ComplexHeatmap (v2.0.0) [43] was used to visualize the expression profiles for the 5249 genes across all experiments.

#### Canonical pathway analysis

Canonical pathway analysis was performed with Ingenuity Pathway Analysis (IPA) version 68752261 (Qiagen Inc.) [44]. The data set was compared to the reference set “Whole Human Genome Microarray 4x44 K”. log_2_FC < -1.0 were considered downregulated, while log_2_FC > 1.0 were considered upregulated. Expr False Discovery Rate (q-value) < 0.001 was considered significant.

#### GO analysis

Gene ontology analysis was performed with Panther Gene Ontology-slim molecular function analysis tool (http://www.pantherdb.org/) and Fisher’s exact test [45, 46]. Differentially expressed genes with log_2_FC < -1.0 were considered downregulated, while genes with log_2_FC > 1.0 were considered upregulated. FDR < 0.05 was considered significant.

### Western blot (WB)

hiPSC-derived astrocytes were collected for WB experiments at week 4 (d + 28) of maturation. Cells were washed in DPBS and harvested in a lysis buffer of 10 ml M-PER (Cat N° 78501), 1 tablet PhosSTOP™ Phosphatase Inhibitor Cocktail (Roche Diagnostics, Cat N° 04906845001) and 1 tablet cOmplete™ Protease Inhibitor (Roche Diagnostics, Cat N° 04693116001). Protein content was measured using a Micro BCA™ Protein Assay kit (Cat N° 23235) per the manufacturer’s instructions. Samples containing 40 μg protein were supplemented with SDS-containing lane marker reducing sample buffer (Cat N° 39000), denatured at 95 °C for 5 min and loaded on NuPAGE® 4–12% Bis–Tris 1.0 mm Mini gel (Cat N° NP0321BOX). The gel was run at a 100 V for 1 h 45 min and transferred to a 0.2 μm nitrocellulose membrane of a Trans-Blot Turbo Mini Transfer Pack (Bio-Rad, Hercules, CA, USA, Cat N° 1704158) using the 7 min, 2.5 A, 25 V program of the Bio-Rad Trans-Blot® Turbo Transfer system. The membrane was blocked in 3% BSA (Sigma-Aldrich, Cat N° A7906) diluted in Tris-buffered saline (Sigma-Aldrich, Cat N° T5912-1L) with Tween (Sigma-Aldrich, Cat N° P1379-500ML) (TBST) for 1 h and subsequently incubated overnight with primary antibodies (see Supplementary Table 1, Additional file 1) in 3% BSA-TBST at 4 °C. The following day, the membrane was washed thrice for 10 min in TBST and incubated with secondary antibodies diluted in TBST for 1 h at RT. Finally, the membrane was washed thrice for 10 min in TBST, treated with Pierce ECL (Cat N° 32106) and imaged with a chemiluminescence ImageQuant LAS4000. Quantifications were made with ImageJ.

### Multiplex array

Astrocyte conditioned medium was collected from each condition after 48 h. Cytokine measurements were obtained using the Meso Scale Discovery (MSD) system. The commercially available human-specific V-PLEX Proinflammatory Panel 1 assay (MSD, K15049G-1) was used. Each sample was measured in technical duplicates and the assay was performed according to the manufacturer’s protocol. Cytokine concentrations were measured using the MESO QUICKPLEX SQ 120 machine from MSD with the software DISCOVERY WORKBENCH 4.0. Only values above detection level and with a CV value below 25 were included in the analysis.

### qPCR

hiPSC-derived astrocyte pellets were collected with accutase and stored at -80 °C for qPCR experiments at week 1–4 of maturation. For total RNA extraction, each cell pellet was dissolved in 1 ml Tri Reagent® (Sigma-Aldrich, Cat N° T9424). The homogenate was supplemented with 200 μl/sample chloroform (Sigma-Aldrich, Cat N° 319988), inverted for 15 s and incubated at RT for 10 min. Next, the sample was centrifuged for 15 min at 12 000 rpm at 4 °C, and the aqueous phase was transferred to new RNase-free tubes and precipitated with 500 μl/sample isopropanol (Merck Millipore, Cat N° 1.09634.2511). After an incubation of 10 min at RT, the sample was centrifuged for 10 min at 12 000 rpm at 4 °C and the supernatant was discarded. The pellet was washed once with 1 ml 75% ethanol (VWR, Radnor, PA, USA, Cat N° 20.821.296), and dissolved in 11 μl RNase-free water. RNA concentrations were determined with NanoDrop 1000 Spectrophotometer V3.8.1. cDNA was synthesized from 1 μg RNA using SuperScript® III First-Strand Synthesis System for RT-PCR (Cat N° 18080–051) per manufacturer’s instructions. qPCR was performed using Fast SYBR™ Green Master Mix (Cat N° 4385612) and TaqMan™ Fast Universal PCR Master Mix (Cat N° 4352042) (for primers see Supplementary Table 1, Additional file 1) on a StepOnePlus Real-Time PCR machine. Relative gene expression was normalized to β-actin and hiPSCs and calculated with the 2^−ΔΔCt^ method [47].

### Live cell calcium recordings of astrocytes

hiPSC-derived astrocytes were plated at 50 000 cells/cm^2^ in 6-well plates at week 5 of maturation and analysed at week 6. Cells were labelled with 5 μM Fluo-4 AM (Cat N° F14201), and spontaneous Fluo-4 fluorescence was recorded over 90 s with a 10X objective using a Nikon A1R TiE confocal microscope equipped with a Plan Apo 10X objective lens and analyses using a customized Nikon script. The script calculated the difference between min and max fluorescence intensity in individual astrocytes during the 90 s recording. The threshold for active cells was set to an intensity increase > 100.

### Scanning electron microscopy (SEM)

Astrocyte, motor neuron and NMJ morphologies were evaluated with SEM at day 28 of the coculture timeline using a previously published method [25, 26]. In brief, cultures were fixed in 2.5% glutaraldehyde (Agar Scientific, Essex, UK, Cat N° R1020) in 0.1 M Na-cacodylate buffer (Sigma-Aldrich, Cat N° C0250) and the SND75 devices were carefully removed from the Aclar sheets. Next, the cultures were incubated in 1% osmium tetroxide (Electron Microscopy Sciences, Cat N° 19150) and dehydrated in a graded ethanol series to 100% ethanol. The sheets were then critical point dried, mounted on SEM support stubs and coated with 5 nm Chromium in a Leica ACE600. Cultures were imaged with a Zeiss Sigma SEM.

### LDH analysis

hiPSC-derived motor neuron-NPCs were plated at day 10 of differentiation at a density of 25 000 cells/cm^2^ and differentiated until day 21. At day 21 of motor neuron differentiation, astrocytes at week 5.5 of maturation (d + 38) were plated on top of the motor neurons in motor neuron medium at a density of 25 000 cells/cm^2^. The cocultures were kept for 14 days in motor neuron medium, and media samples were collected on days 2, 7 and 14. Monocultured day 21 motor neurons were incubated with 100 μM Arsenite treatment for 48 h as a positive control for cell death. Samples were analysed CyQUANT™ LDH Cytotoxicity Assay Kit (Cat N° C20300) according to the manufacturer’s instructions.

### Neurite network quantifications

On days 28 and 35 of coculture (1 and 2 weeks after adding astrocytes, respectively), z-stack tile scan confocal images were taken at 10X magnification of the motor neuron/astrocyte compartment using a Nikon A1R confocal microscope equipped with a Plan Apo 10X objective lens. Images were cropped to display only the channel of the device, and motor neuron neurite (NEFH) volume was quantified in 3D using a customized Nikon software script.

### Neurite outgrowth quantifications

Neurite outgrowth was analysed as previously described [25]. On days 28 and 35 of coculture (1 and 2 weeks after adding astrocytes, respectively), tile scan confocal images were taken at 10X magnification of the NEFH fluorescence in the myotube compartment using an inverted Leica SP8 DMI8 confocal microscopy equipped with a HC PL APO CS2 10x/0.40 dry objective lens. Motor neuron neurites were isolated using ilastik 1.3.3post1 Pixel Classification software [48], and the number of pixel intersections was calculated per intersection line using an ImageJ 1.52p software linear Scholl analysis script.

### Live-cell calcium recordings of NMJs

Recordings were made using a previously described protocol [25, 26]. On day 28 of the coculture timeline (1 week after adding astrocytes), myotubes were labelled with 5 μM Fluo-4 AM. The motor neuron/astrocyte compartment was stimulated with 50 mM potassium chloride (KCl) and Fluo-4 fluorescence was recorded in the myotubes using a Nikon A1R confocal microscope equipped with a Plan Apo 10X objective lens and analysed with NIS-Elements AR 4.30.02 software’s Time Measurement tool. The myotube compartment was stimulated directly with 50 mM KCl, to calculate the percentage of motor neuron-stimulated myotubes through functional NMJs of the total number of active myotubes.

### Quantification and statistical analysis

Statistical analyses were made with GraphPad Prism 9.2.0, where data were tested for normal Gaussian distribution using the D’Agostino-Pearson omnibus normality test, Anderson–Darling test, and Shapiro–Wilk normality test. Statistical details of experiments can be found in the figure legends. For differences of mean between two groups, unpaired t test or Mann–Whitney were used, while one/two-way ANOVA with Tukey’s multiple comparisons test or Kruskal–Wallis test with Dunn’s multiple comparisons test were used for difference of means between multiple groups. *p < 0.05, **p < 0.01, ***p < 0.001, and ****p < 0.0001. Each experiment included at least 3 biological replicates, where a biological replicate represents an independent differentiation from hiPSCs to terminal cell type, an independent myoblast differentiation into myotubes or an independent coculture in a one-compartment plate or microfluidic device.

## Results

### hiPSCs differentiate into functional astrocytes independent of *FUS* mutations

**Fig. 1:**
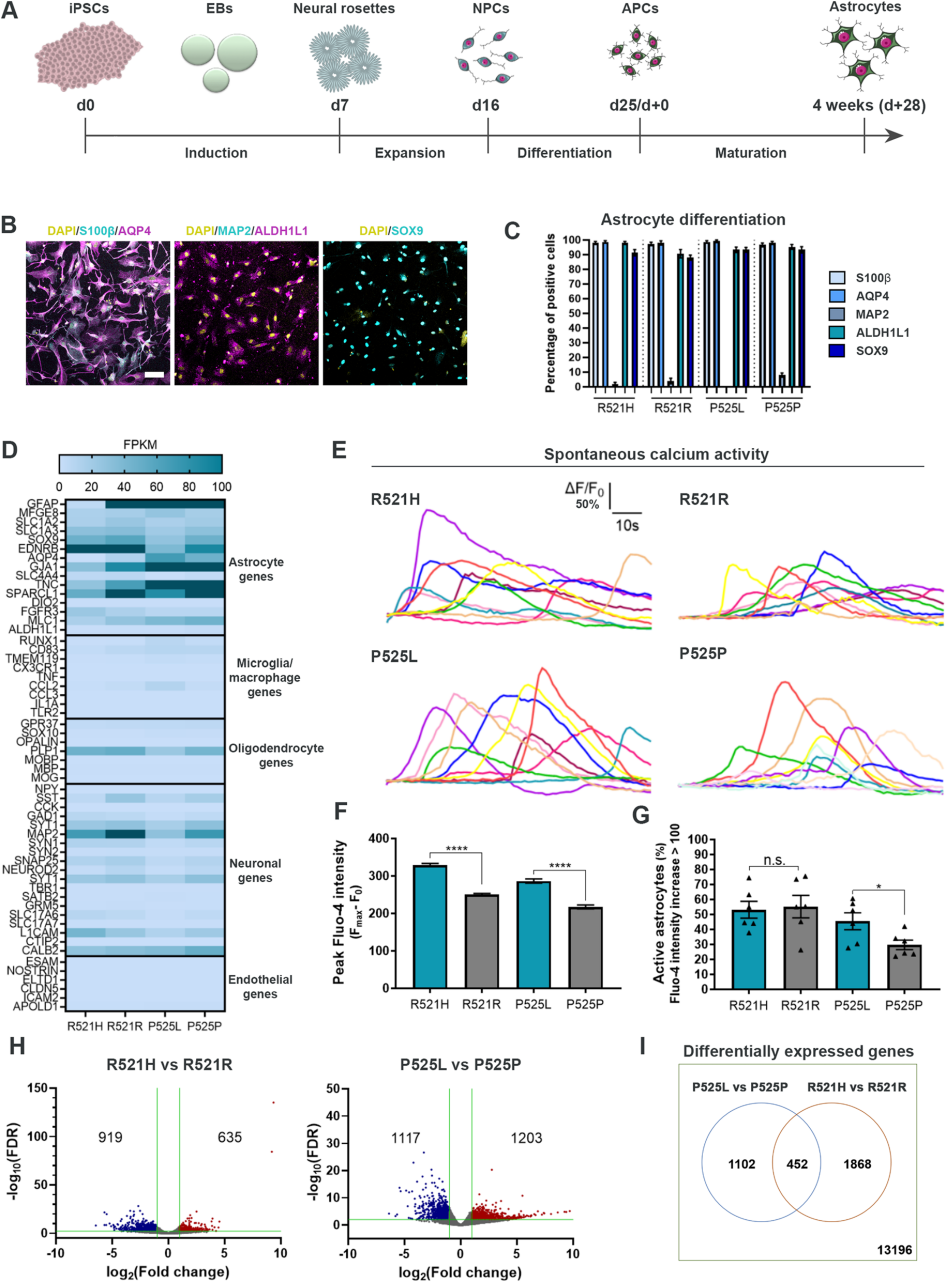
Generation and characterisation of functional hiPSC-derived astrocytes. **A** Overview of the astrocyte differentiation protocol. hiPSCs were dissociated at day 0 (d0) and cultured for the induction phase as embryoid bodies (EBs) before they were plated for neural rosette formation at day 7 (d7). The neural rosettes underwent expansion to generate neural progenitor cells (NPCs) until day 16 (d16). NPCs were then differentiated into astrocyte progenitor cells (APCs, d25/d+0) after which the APCs were matured into astrocytes for an additional 4 weeks (d + 28). **B** Representative confocal images of astrocytes at week 4 (d + 28) of maturation stained with astrocytic markers S100β, AQP4, ALDH1L1 and SOX9 in addition to neuronal marker MAP2. Nuclei stained with DAPI. Scale bar: 75 μm. **C** Quantification of the number of cells positive for astrocyte and neuronal markers. Mean ± s.e.m. of 3 biological replicates (*n* = 15 images). **D** Heatmap of the expression of cell type-specific genes in 4 weeks mature astrocytes from RNAseq experiment performed in 3 biological replicates. Dark blue cells: FPKM > 100. **E** Functional assessment of spontaneous calcium transients in 6 weeks mature astrocytes loaded with the Fluo-4 dye. Y-axis range: ΔF/F_0_ = 50%. **F** Quantification of peak Fluo-4 intensity. Mann–Whitney test. ****p < 0.0001. **G** Percentage of active astrocytes. Inactive astrocyte cut-off: Fluo-4 intensity increase < 100. Unpaired t test. *p < 0.05. **H** Volcano-plots of up- (red) and down- (blue) regulated genes in week 4 mature astrocytes. Green lines indicate thresholds: log_2_FC < -1.0 with –log_10_(FDR) > 2.0 were considered downregulated, and log_2_FC > 1.0 with –log_10_(FDR) > 2.0 were considered upregulated. **I** Comparison of differentially expressed genes between mutation sets. Graphs in panels (**F** and **G**) show mean ± s.e.m. of 3 biological replicates with each 2–6 technical replicates. Cell illustrations are modified from Smart Servier Medical Art licensed under a Creative Commons Attribution 3.0 Unported License (https://creativecommons.org/licenses/by/3.0/). See also Suppl. Figure 1–3, Additional file 2, as well as Additional files 3, 4, 5 and 6

To evaluate the role of astrocytes in ALS, we differentiated hiPSCs from two ALS-patients with *FUS* mutations (R521H and P525L) and their corresponding CRISPR-Cas9 gene-edited isogenic controls (R521R and P525P) [25, 27, 28] into astrocytes using a slightly modified version of a recently published protocol [29]. The P525L mutation causes an aggressive juvenile form of ALS, while the more common R521H mutation causes adult-onset ALS [27], and the inclusion of both lines demonstrate a broad disease-onset spectrum. The astrocyte differentiation (25 days) generated APCs, which were further matured into astrocytes during 4 weeks (Fig. 1A). The differentiation efficiency was evaluated with bright-field microscopy (Suppl. Figure 1A, Additional file 2), qPCR (Suppl. Figure 1B, Additional file 2), ICC (Fig. 1B-C, Suppl. Figure 2–3, Additional file 2) and bulk RNAseq (Fig. 1D, Suppl. Figure 1C-D, Additional file 2). During the 4 weeks of maturation, an increase in both gene (Suppl. Figure 1B, Additional file 2) and protein (Suppl. Figure 2, Additional file 2) expression of astrocyte markers S100 calcium-binding protein β (S100β) and AQP4 were observed. In addition, the astrocytes stained increasingly positive for astrocyte-specific markers Aldehyde Dehydrogenase 1 Family Member L1 (ALDH1L1) and SRY-Box Transcription Factor 9 (SOX9), while the neuronal marker Microtubule-Associated Protein 2 (MAP2) remained at a low expression level (Suppl. Figure 2–3, Additional file 2). After 4 weeks of maturation, approximately 95% of astrocytes were positive for all astrocyte markers (Fig. 1B-C) showing no difference in the differentiation potential between patient and gene-repaired hiPSC-lines. Additionally, RNAseq revealed a high expression level of astrocyte-specific genes in comparison to genes specific for other cell type in the central nervous system (Fig. 1D). To assess the functionality, we performed live-cell recordings of spontaneous calcium transients using Fluo-4 (Fig. 1E-G). All four astrocyte lines showed characteristic calcium transients demonstrating clear functionality (Fig. 1E, Additional files 3, 4, 5 and 6). Both mutant astrocyte populations displayed higher peak intensities in comparison to controls (Fig. 1F) suggesting mutant *FUS*-mediated calcium transient hyperactivity. In addition, P525L also revealed an increase in the number of active astrocytes (Fig. 1G). Next, we looked at the differences in gene expression between *FUS*-ALS and isogenic control astrocytes. Our principal component analysis demonstrated minor variations between biological replicates (Suppl. Figure 1C, Additional file 2) and a clustering of controls, while R521H and P525L astrocytes showed an almost opposite gene expression profile (Suppl. Figure 1D, Additional file 2). Overall, 635 and 1203 genes were shown upregulated in mutant R521H and P525L astrocytes, respectively, while 919 (R521H) and 1117 (P525L) genes were downregulated in comparison to their respective controls (Fig. 1H). Of all recorded genes, 452 differentially expressed genes overlapped between the two gene-pairs, while 1102 (P525L vs P525P) and 1868 (R521H vs R521R) were unique to the individual mutation (Fig. 1I). In addition, we compared our differential gene expression with previously published transcriptomic data sets from hiPSC-derived astrocytes generated from *VCP*, *FUS*, *SOD1* and *C9orf72* ALS patients (Suppl. Figure 1E, Additional file 2) [42, 49]. Interestingly, we observed some similarity in the gene expression between our P525L astrocytes, the *SOD1*^*D90A*^ and the *C9orf72* patient astrocytes, while the R521H astrocytes had strikingly opposite gene expression profile. *VCP*^*R155C*^ and *FUS*^*H517Q*^ were shown to range in between P525L and R521H expression levels. Generally, a large variation in the differential gene expression across lines could be appreciated. Taken together, these results confirm that we are able to generate pure cultures containing functionally active astrocytes from each hiPSC-line independent of the presence of a *FUS* mutation.

### *FUS*-ALS astrocytes display increased reactivity

**Fig. 2:**
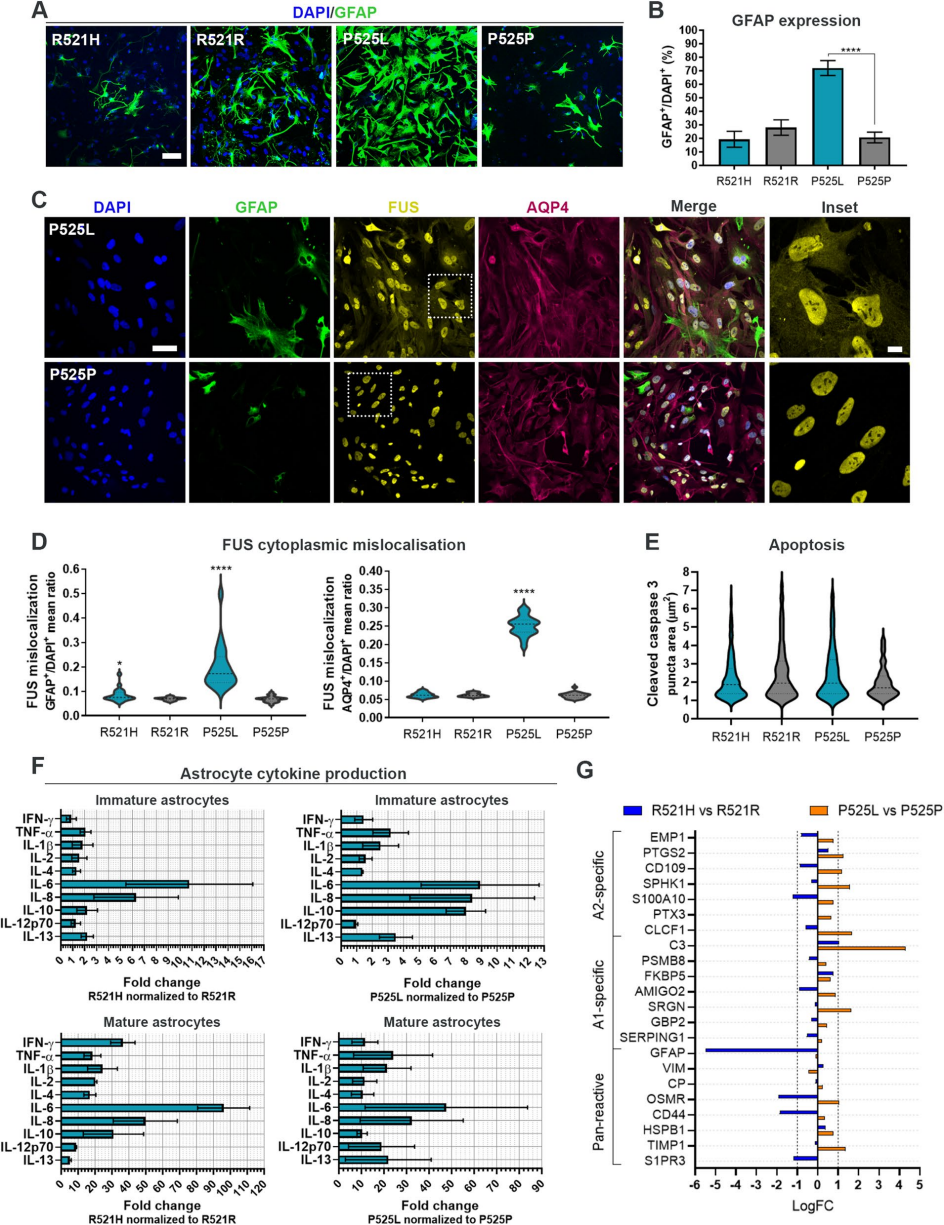
*FUS-*ALS astrocytes display increased reactivity. **A** Confocal images of GFAP expression in 4 weeks mature astrocytes. Nuclei stained with DAPI. Scale bar: 75 μm. **B** Quantification of GFAP expression. One-way ANOVA with Tukey’s multiple comparisons test. Mean ± s.e.m. of 3 biological replicates (*n* = 15 images). ****p < 0.0001. **C** Representative confocal images of FUS protein localisation in 4-week mature P525L and P525P astrocytes. Nuclei stained with DAPI. Scale bar: 50 μm. Inset scale bar: 10 μm. **D** Violin plot of FUS cytoplasmic mislocalisation in GFAP^+^ and AQP4^+^ astrocytes. Data from 3 biological replicates (*n* = 60 images). Unpaired t test and Mann–Whitney test, respectively. *p < 0.05 and ****p < 0.0001. **E** Apoptotic assessment in week 4 mature astrocytes stained with cleaved caspase 3. Violin plot from 3 biological replicates (*n* = 30 images) with outliers (Q = 0.1%) removed. **F** Secretome analysis of immature (d25/d + 0) and more mature (week 4) astrocytes. Mean ± s.e.m. of 3 biological replicates. **G** RNAseq reactive astrocyte gene expression profiling of week 4 mature astrocytes. See also Suppl. Figure 3–4, Additional file 2

Glial fibrillary acidic protein (GFAP) is a widely used astrocyte marker but also indicative of astrocyte reactivity [50]. Since an increased GFAP expression has been reported in *post-mortem* samples from ALS patients [12], we investigated the expression of GFAP within our astrocyte populations using ICC. Our analysis showed a progressive increase in GFAP during astrocyte maturation in all hiPSC-lines (Suppl. Figure 3A-B, Additional file 2), however, especially P525L astrocytes demonstrated a significantly higher percentage of GFAP-positive cells in the population compared to the isogenic control (Fig. 2A-B). These findings were confirmed with Western blot (WB) (Suppl. Figure 3D, Additional file 2), and also our qPCR analysis presented a maturation-dependent increase in *GFAP* gene expression (Suppl. Figure 3E, Additional file 2).

As we previously demonstrated cytoplasmic mislocalisation of FUS protein in *FUS*-ALS patient fibroblasts, hiPSCs and hiPSC-derived motor neurons [27], we also evaluated the cellular localisation of FUS protein within our astrocytes. In line with these findings, both *FUS*-mutant lines showed a cytoplasmic mislocalisation of FUS in the GFAP-positive astrocyte population, while only P525L astrocytes displayed mislocalisation within the more general AQP4-positive population (Fig. 2C-D). No difference in apoptosis as measured by the size of cleaved caspase 3 ICC punctae was observed (Fig. 2E).

ALS astrocytes secrete toxic factors [20, 21, 23], so to investigate this further, we performed a secretome analysis by measuring the presence of 10 inflammatory cytokines (IFN-γ, TNF-α, IL-1β, IL-2, IL-4, IL-6, IL-8, IL-10, IL-12p70 and IL-13) in the media from immature (APCs d25/d+0) and more mature astrocytes (maturation week 4) (Fig. 2F). Remarkably, immature R521H astrocytes showed a high secretion of especially IL-6 and IL-8, while P525L astrocytes showed an increased secretion of the majority of cytokines at this early differentiation state. In more mature mutant astrocytes, the cytokine secretion increased several tenfolds, which signifies that the inflammatory response of astrocytes is differentiation-dependent (Suppl. Figure 4A, Additional file 2). Both IL-4 and IL-10 are involved in suppressing/regulating the immune response [51], and their presence in the *FUS*-ALS secretome might therefore also indicate an astrocytic attempt to counteract the other pro-inflammatory cytokines. To explore this further we performed a gene-ontology (GO) analysis on our RNAseq data to examine both up- and downregulated molecular functions (Suppl. Figure 4B, Additional file 2). In R521H astrocytes, no upregulated pathways were found, however, several biological pathways correlating with cytokine functions (cytokine activity, cytokine receptor activity and cytokine binding) were downregulated, which supports our hypothesis of an auto-regulatory attempt to counteract the inflammatory response. Interestingly, the P525L astrocytes showed an upregulation of both cytokine receptor binding and cytokine activity, which argues for a mutation- and/or age of disease onset-dependent reactive effect.

Since our data so far favoured a toxic reactive phenotype in our *FUS*-ALS astrocytes, we turned our attention to a previously suggested binary categorisation of reactive astrocytes; “A1” astrocytes being neurotoxic and “A2” astrocytes being neuroprotective [52]. Our results disclosed neither a clear A1 nor A2 gene expression profile in our *FUS* mutant astrocytes in comparison to controls but showed both an up- and downregulation of genes within each group as well as for genes present in pan-reactive astrocytes (Fig. 2G). Taken together, these results demonstrate a *FUS*-mutant-mediated astrocyte reactivity, which affects the transcriptome and secretome in a toxic although heterogeneous mutation-dependent manner.

### hiPSC-derived astrocytes are successfully integrated in a microfluidic model of the human motor unit

**Fig. 3:**
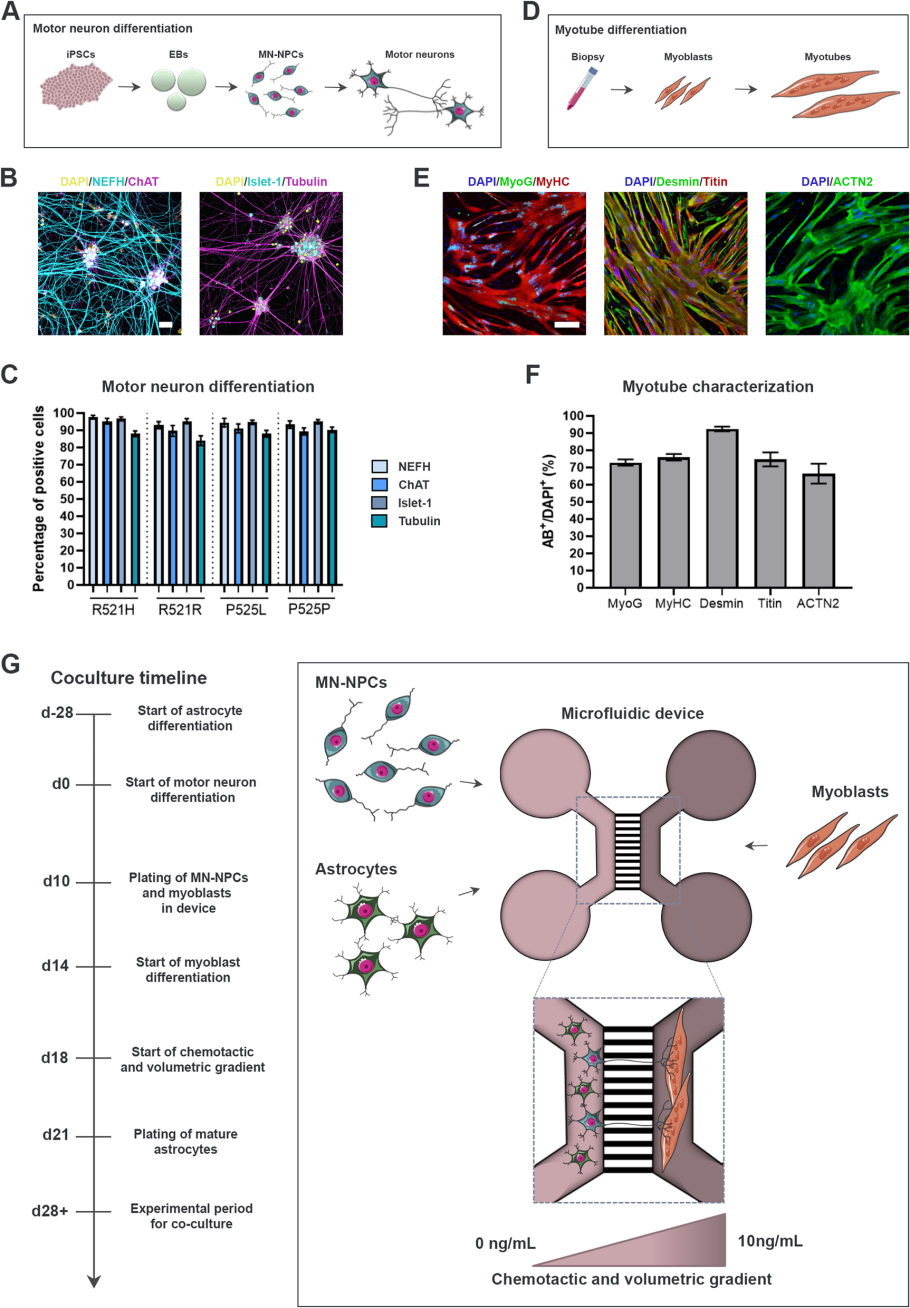
Motor neuron and myotube verification for the establishment of microfluidic coculture model. **A** Overview of motor neuron differentiation from hiPSCs through an EB state towards MN-NPCs and finally post-mitotic spinal motor neurons. **B** Representative confocal images of mature motor neurons at day 28 of differentiation stained with motor neuron markers ChAT and Islet-1 in addition to pan-neuronal markers NEFH and βIII-tubulin (Tubulin). Nuclei stained with DAPI. Scale bar: 25 μm. **C** Quantification of the number of cells positive for neuronal markers. Mean ± s.e.m. of 3 biological replicates (*n* = 15 images). Kruskal–Wallis test with Dunn’s multiple comparisons test. **D** Overview of myotube differentiation. Primary human myoblasts were isolated from vastus lateralis muscle, expanded and differentiated into multinucleated elongated myotubes. **E** Representative confocal images of myotubes stained with markers (AB^+^) MyoG, MyHC, desmin, titin and ACTN2. Nuclei stained with DAPI. Scale bar: 200 μm. **F** Quantification of myogenic markers in multinucleated myotubes. Mean ± s.e.m. of 3 biological replicates (*n* = 15 images). **G** Schematic overview of coculture between hiPSC-derived astrocytes and motor neurons with human primary myoblast-derived myotubes in a compartmentalized microfluidic device. A chemotactic and volumetric gradient is established to promote motor neuron neurite polarization [25, 26]. Experiments were conducted 1 week (d28) and 2 weeks (d35) after plating the mature astrocytes. Cell illustrations are modified from Smart Servier Medical Art licensed under a Creative Commons Attribution 3.0 Unported License (https://creativecommons.org/licenses/by/3.0/). See also Suppl. Figure 5, Additional file 2

To evaluate the effect of astrocytes in a novel multicellular model, we optimised our previously established protocol for the generation of a human motor unit in microfluidic devices containing functional NMJs [26]. hiPSC-derived motor neurons were differentiated from each hiPSC-line (Fig. 3A) using our formerly established protocol [27], and the differentiation potential was evaluated with ICC (Fig. 3B, Suppl. Figure 5A-B, Additional file 2). At day 28 of motor neuron differentiation, approximately 85–95% of cells stained positive for motor neuron-specific markers choline acetyltransferase (ChAT) and Islet-1 in addition to the pan-neuronal markers neurofilament heavy chain (NEFH) and βIII-tubulin (Tubulin) with no difference between hiPSC-lines (Fig. 3C). A limitation of our previous motor unit model was the restricted viability of the mesoangioblast-derived myotubes [26], so to prolong the coculture, we instead made use of primary human myoblasts (Fig. 3D). The myoblasts ability to differentiate and fuse into multinucleated myotubes was evaluated with ICC (Fig. 3E, Suppl. Figure 5C-D, Additional file 2), and showed 70–90% expression of myotube-markers myogenin (MyoG), myosin heavy chain (MyHC), desmin, titin and α-actinin (ACTN2) (Fig. 3F). For the coculture in compartmentalized microfluidic devices, day 10 motor neuron-neural progenitor cells (MN-NPCs) were plated in the compartment at one side of the microgrooves, while myoblasts were plated in the opposite compartment (coculture timeline shown in Fig. 3G). Myoblasts were differentiated into myotubes, and a chemotactic and volumetric gradient was established to promote the motor neuron neurite across-microgroove migration in order for motor neurons and myotubes to interact and form NMJs [26]. Week 3.5 mature astrocytes were subsequently plated on top of the maturing motor neurons in the motor neuron compartment of the microfluidic device and the cocultures were evaluated 1 week (day 28) and 2 weeks (day 35) later.

### *FUS*-ALS astrocytes fail to integrate in the motor neuron network

**Fig. 4:**
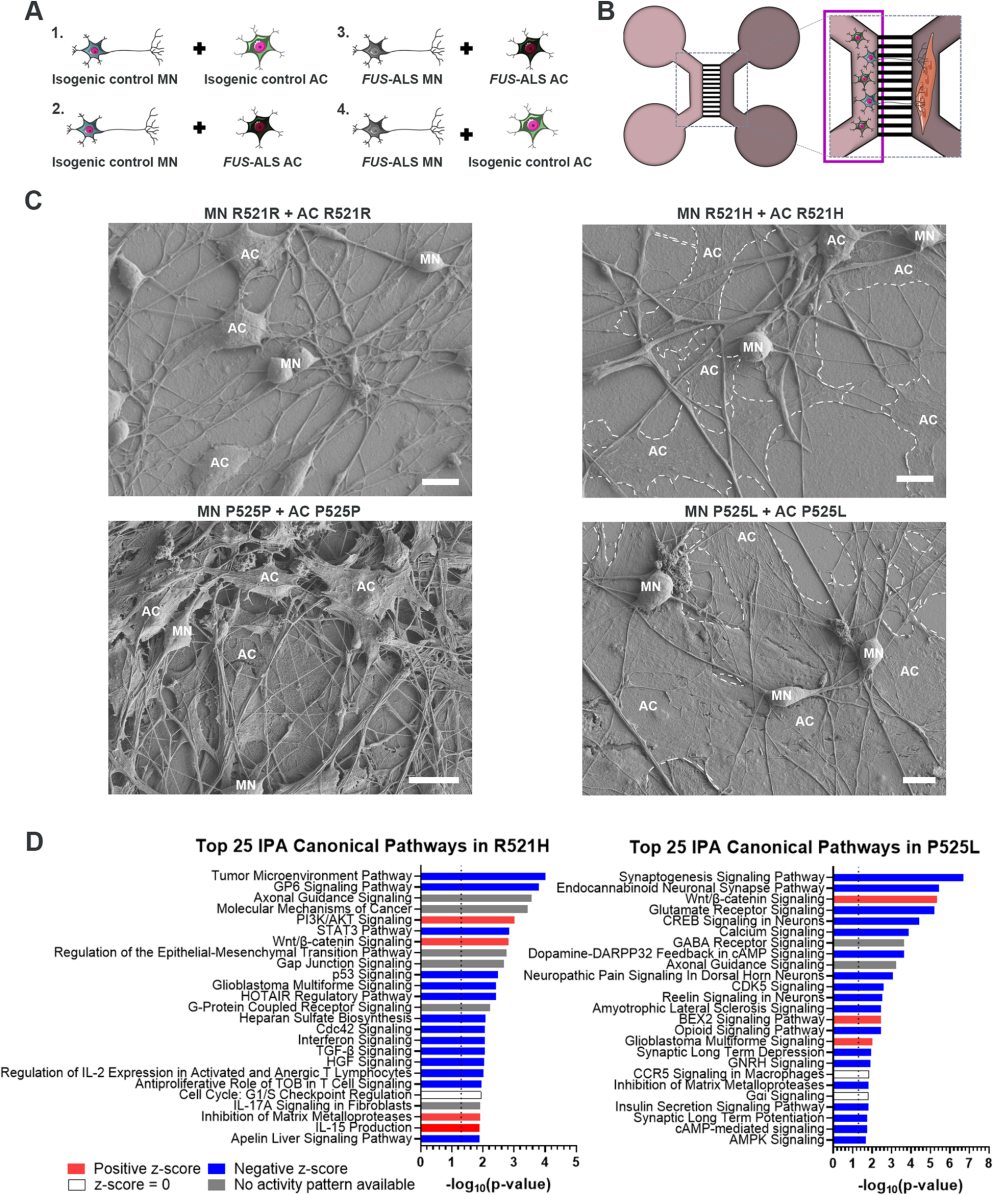
*FUS*-ALS astrocytes fail to integrate in the motor neuron network. **A** Overview of coculture setup between hiPSC-derived motor neurons (MN) and astrocytes (AC). **B** Experimental assessment was performed in the highlighted motor neuron/astrocyte compartment of the microfluidic device after 1 and 2 weeks of coculture. **C** Scanning electron microscopy images of motor neuron and astrocyte cocultures in microfluidic devices after 1 week. Mutant astrocytes are circled with a white dashed line. Scale bar: 10 μm. **D** Top 25 RNAseq canonical pathways in mutant astrocytes compared to controls at week 4 of maturation. Analysis was performed with a cut-off Log Ratio of -1.0 to 1.0 and an FDR of 0.001. Dotted line marks *p*-value = 0.05. Cell illustrations are modified from Smart Servier Medical Art licensed under a Creative Commons Attribution 3.0 Unported License (https://creativecommons.org/licenses/by/3.0/). See also Suppl. Figure 6, Additional file 2

Four coculture conditions were established to assess the effect of astrocytes on the motor neurons; fully-isogenic control (IC) (IC motor neurons and IC astrocytes), fully-mutant (mutant motor neurons and mutant astrocytes) and combined setups (Fig. 4A-B). Interestingly, we observed an increase in GFAP expression in the astrocytes in all conditions, which suggests that the presence of motor neurons further activates the astrocytes independently of *FUS* mutations (Suppl. Figure 6A, Additional file 2). Overall, the astrocytes displayed a more elongated morphology and appeared well-integrated in the motor neuron networks in the fully-IC conditions as well as for the combined setup of mutant motor neurons and IC astrocytes (Suppl. Figure 6A, Additional file 2). In contrast, the *FUS*-ALS astrocytes appeared hypertrophic and non-integrated in the fully-mutant conditions as well as in the combined setup of IC motor neurons and mutant astrocytes. These results were confirmed using scanning electron microscopy (SEM), which showed that the mutant R521H and P525L astrocytes generally appeared large, flat and non-engaging with the motor neurons (Fig. 4C). The presence of the *FUS-*mutant astrocytes had no apparent influence on the motor neuron network, as we did not measure any difference in motor neuron neurite volume in the motor neuron/astrocyte compartment across conditions at either time point (Suppl. Figure 6B-G, Additional file 2). To investigate whether the astrocyte presence caused motor neuron death, we performed a Lactate Dehydrogenase (LDH) activity assay (Suppl. Figure 6H-I, Additional file 2). The R521H astrocytes had an initial mild cytotoxic effect on both R521R and R521H motor neurons after 2 days of coculture. However, this small effect disappeared over the course of the 14 days coculture, and was neither comparable to the 100 μM arsenite-treated motor neurons, where 100% cell death was observed (data not shown). No significant differences in cell death were measured in the P525L and P525P conditions.

To further assess the mechanisms involved in *FUS*-ALS astrocyte function, we performed an IPA canonical pathway analysis on our RNAseq data. The majority of pathways were downregulated in *FUS*-ALS astrocytes representing a loss-of-support mechanism since these pathways are largely involved in the maintenance of neuronal homeostasis (Fig. 4D). Notably, finding the synaptogenesis signaling pathway and the axonal guidance signaling pathway in the top 3 of P525L and R521H respectively further supported the dysregulation of astrocyte-neuronal interaction. Collectively, our data demonstrate that *FUS*-ALS astrocytes fail to integrate and support the motor neuron network.

### P525L *FUS*-ALS astrocytes impair motor neuron neurite outgrowth

**Fig. 5:**
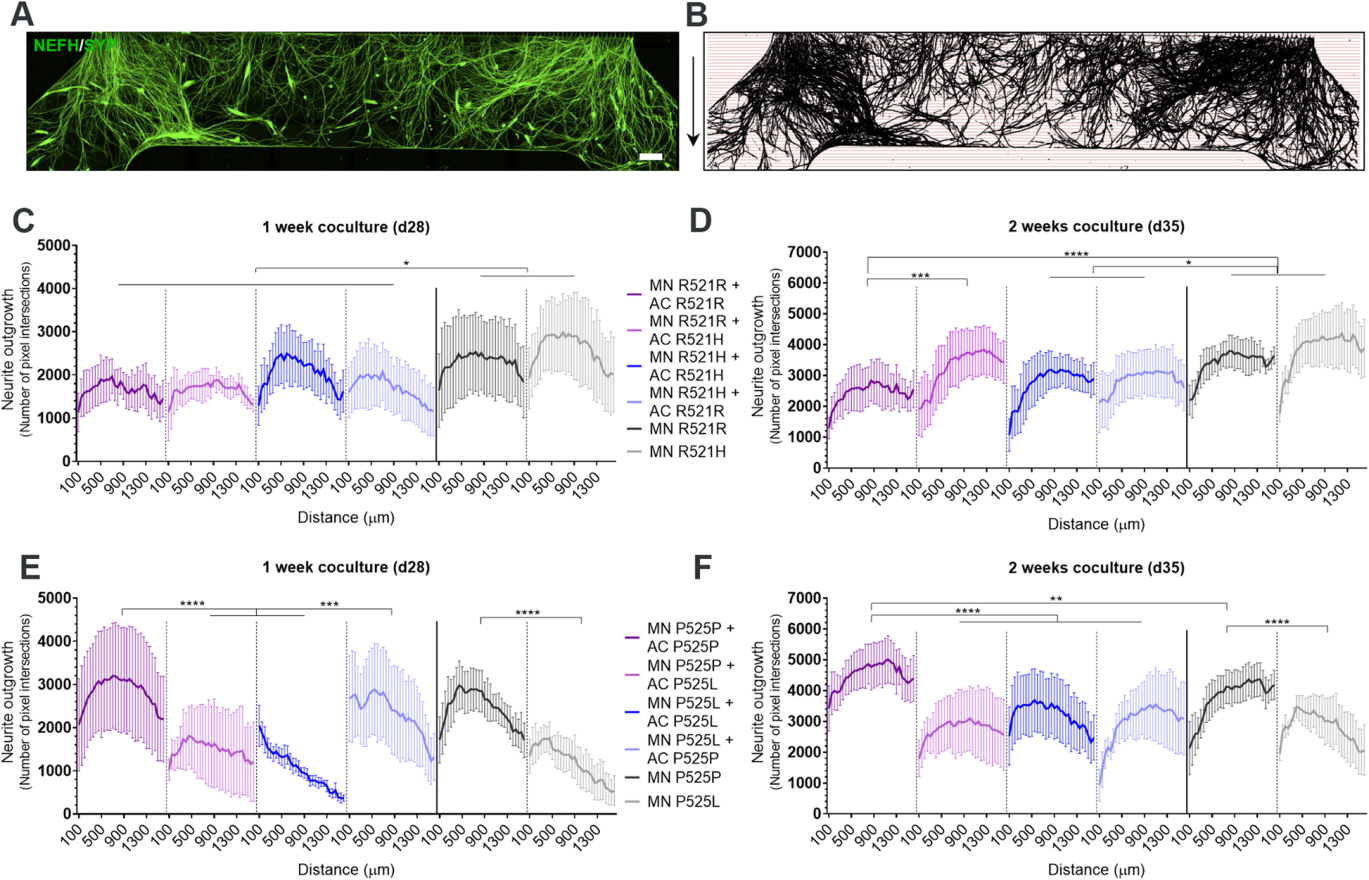
P525L *FUS*-ALS astrocytes impair motor neuron neurite outgrowth. **A** Example of a tile scan confocal image of motor neuron (MN) neurite (NEFH/SYP) outgrowth in the myotube compartment after 1 week (d28) of coculture with astrocytes (AC). Arrows (right) depict the neurite growth direction upon exit of the microgrooves. Scale bar: 300 μm. **B** Mask of tile scans with intersection lines at every 50 μm starting from the exit of the microgrooves. **C-F** Neurite outgrowth quantifications of the number of pixel intersections after 1 week (panel **C** + **E**) and 2 weeks (panel **D** + **F**) of coculture between motor neurons, myotubes and astrocytes. Graphs in (panel **C**-**F)** show mean ± s.e.m. of 3 biological replicates. Overall comparisons between genotypes were performed with two-way ANOVA with Tukey’s multiple comparisons test. *p < 0.05, **p < 0.01, ***p < 0.001 and ****p < 0.0001

We previously showed that P525L hiPSC-derived motor neurons have a reduced neurite outgrowth in microfluidic devices in comparison to controls [25]. As we found a predominant downregulation of neuronal support mechanisms with the axonal guidance signaling pathway ranking high in the transcriptomic data from our *FUS*-ALS astrocytes, we sought to evaluate whether the astrocytes could have an effect on the motor neuron neurite outgrowth. Therefore, we performed the same linear Scholl analysis on the motor neuron neurites in the myotube compartment on each coculture condition (Fig. 5A-B). For R521H and R521R setups, we only observed an increase in outgrowth in R521R motor neurons cocultured with mutant R521H astrocytes compared to fully IC conditions after 2 weeks (Fig. 5C-D). These findings confirm that the initial cell death observed in the cocultures has no influence on the long term neurite outgrowth. Interestingly, R521H and R521R cultures without astrocytes collectively showed a larger neurite outgrowth than cocultures including astrocytes, however, no difference was found between the mutant and isogenic motor units, which confirms our previous findings [25]. For the P525L and P525P conditions, we observed a reduced neurite outgrowth in fully-mutant cocultures, combined IC motor neuron and mutant astrocyte conditions as well as in the control motor neuron conditions without astrocytes after 1 week (Fig. 5E). These results demonstrate that P525L astrocytes do not exacerbate the reduced outgrowth of P525L motor neurons, which is already seen without the presence of astrocytes, but that they do limit the outgrowth of control P525P motor neuron neurites. Interestingly, P525P astrocytes rescued this effect on the P525L motor neurons after 1-week coculture but were unable to sustain this influence after 2 weeks. P525P astrocytes were likewise able to increase the outgrowth of P525P motor neurons above the level of P525P motor neurons cultured without astrocytes (Fig. 5F). After 2 weeks of coculture, the differences became less pronounced due to the overall increase in neurite outgrowth across weeks (Fig. 5F), which indicates that the motor neuron neurites are able to recover despite the toxic effects of the mutant astrocytes.

### *FUS*-ALS astrocytes impair the formation and functionality of NMJs

**Fig. 6:**
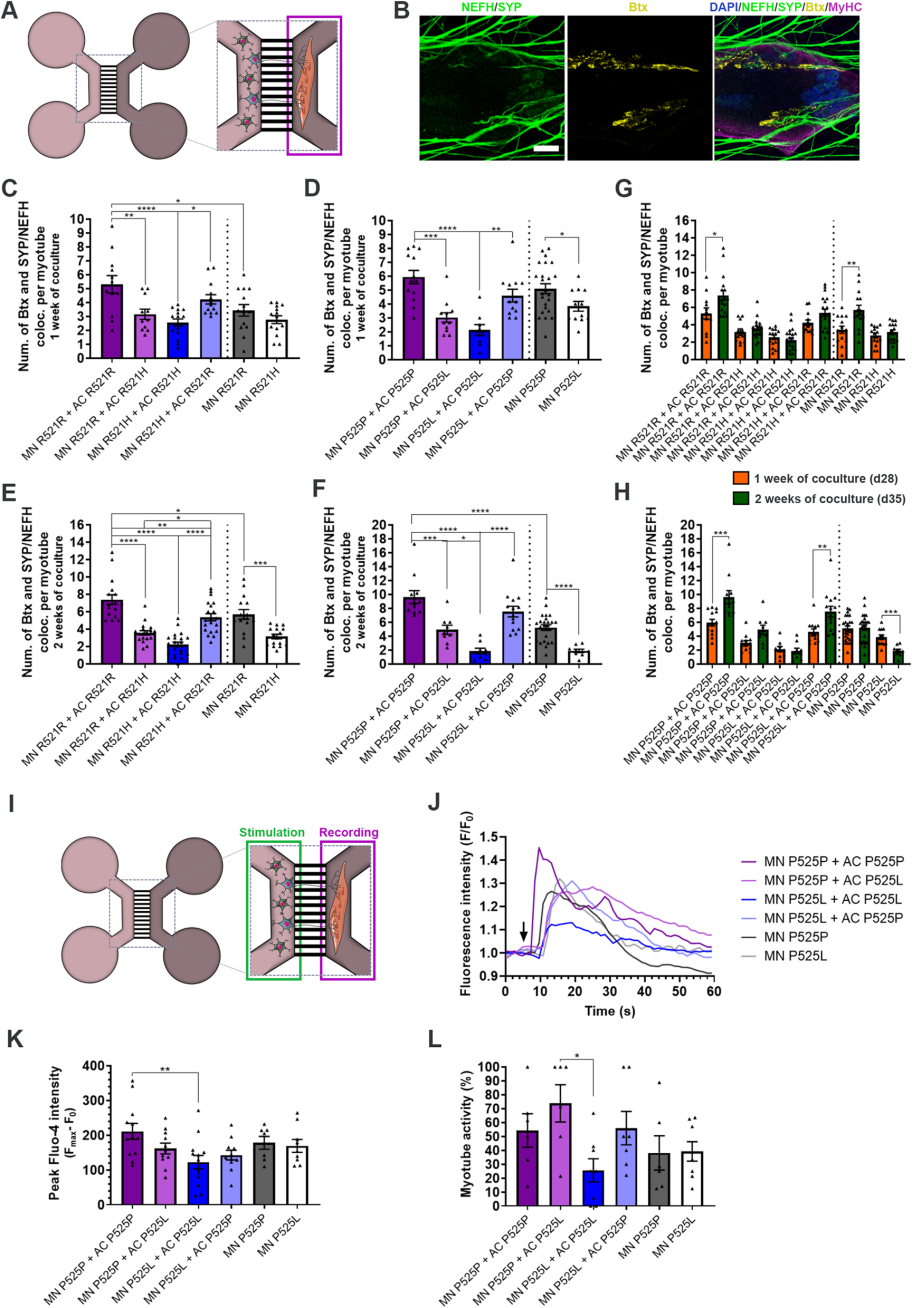
*FUS-*ALS astrocytes impair NMJ formation and functionality. **A** Experimental assessment was performed in the highlighted myotube compartment of the microfluidic device after 1 and 2 weeks of coculture between motor neurons (MN) and astrocytes (AC). **B** Confocal image example of an NMJ. NMJs were identified through colocalisation between motor neuron (NEFH) and presynaptic (SYP) markers with postsynaptic AChR marker (Btx) on MyHC-stained myotubes. Scale bar: 20 μm. **C-F** Quantification of the number of NMJs per myotube after 1 week (panel **C**-**D**) and 2 weeks (panel **E**–**F**) of cocultures between motor neuron/myotubes and astrocytes. **G-H** Number of NMJ formations over time. Graphs in panel **C**-**H** show mean ± s.e.m. of 3 biological replicates. One-way ANOVA with Tukey’s multiple comparisons test (panel **C**-**F**) and unpaired t test (panel **G**-**H**). *p < 0.05, **p < 0.01, ***p < 0.001 and ****p < 0.0001. **I** Schematic overview of live-cell calcium recordings to assess NMJ functionality. The motor neuron/astrocyte compartment was stimulated with 50 mM KCl to evoke an intracellular response, after which an influx in calcium was recorded in myotubes labelled with calcium-sensitive Fluo-4 dye. **J** Representative calcium influx curves in myotubes after KCl stimulation (arrow). **K** Quantifications of peak Fluo-4 intensity. Outliers (Q = 1%) removed. **L** Percentage of NMJ-excitable myotubes of total active myotubes. Data in (**K** and **L**) represent mean ± s.e.m. of 4–5 biological replicates with 2 technical replicates in each experiment. One-way ANOVA with Tukey’s multiple comparisons test. *p < 0.05 and **p < 0.01. Cell illustrations are modified from Smart Servier Medical Art licensed under a Creative Commons Attribution 3.0 Unported License (https://creativecommons.org/licenses/by/3.0/). See also Suppl. Figure 7–8, Additional file 2, as well as Additional files 7, 8, 9, 10, 11, 12 and 13

Next, we assessed the effect of astrocytes on the NMJ formation in the myotube compartment of the microfluidic device (Fig. 6A). NMJ formation was evaluated with ICC and SEM (Fig. 6B, Suppl. Figure 7A, Additional file 2), and the co-localisation between neuritic (NEFH) and presynaptic (synaptophysin (SYP)) markers with acetylcholine receptor (AChR) marker α-bungarotoxin (Btx) was counted per MyHC-labelled myotube (Fig. 6B, Suppl. Figure 7B-D, Additional file 2). Our analysis revealed that *FUS*-mutant astrocytes reduced the formation of NMJs in both fully-mutant and combined IC motor neuron with mutant astrocyte cocultures after 1 week (Fig. 6C-D), and that this negative effect was sustained after 2 weeks of coculture (Fig. 6E-F). In contrast, IC astrocytes had a beneficial effect on the NMJ numbers, which increased in both fully-IC cocultures above the control condition of IC motor units without astrocytes, as well as completely or partially rescued the NMJ formation in combined mutant motor neuron and IC astrocyte conditions. From week 1 to week 2 of the coculture timeline, NMJ numbers increased in fully-IC cocultures, which confirms the continued development of connections between motor neurons and myotubes as the system matures (Fig. 6G-H). This effect was also seen in the R521R motor unit culture without astrocytes (Fig. 6G), as well as in the combined P525L motor neurons and P525P astrocytes (Fig. 6H). Remarkably, we saw a time-dependent decrease in NMJ formation in the P525L motor units without astrocytes, which demonstrates a direct loss of NMJs. Additionally, we quantified the percentage of innervated myotubes and found a reduction in both fully mutant systems after 2 weeks of coculture (Suppl. Figure 8, Additional file 2).

To assess NMJ functionality, we performed live-cell calcium recordings on P525L and P525P conditions as described before [26]. By chemically stimulating the motor neuron/astrocyte compartment with potassium chloride (KCl), we evoked an influx of calcium in Fluo-4 labelled myotubes, confirming transmission through a functional NMJ connection (Fig. 6I, Additional files 7, 8, 9, 10, 11, 12 and 13). Influx curves could be recorded in all conditions (Fig. 6J), and a reduction in peak size in the fully mutant system compared to fully isogenic cocultures could be appreciated (Fig. 6K). Similarly, a reduction in the percentage of NMJ-excitable myotubes were found in the fully mutant systems (Fig. 6L), while a rescuing trend could be observed in P525L motor units cocultured with P525P astrocytes. No obvious difference was observed between the P525P and P525L cocultures without astrocytes, which indicates that the difference in NMJ formation (Fig. 6D) could be explained by a larger presence of immature non-functional NMJs in P525P motor unit systems. In addition, this could also explain the lack of change in NMJ numbers in P525P motor units between week 1 and week 2 cocultures, as the system might have reached its maximum potential for NMJ formation (Fig. 6H).

Taken together, these results demonstrated that *FUS*-ALS and especially P525L astrocytes impair motor neuron neurite outgrowth, NMJ formation and functionality, while IC astrocytes are able to rescue many of these aberrations.

### *FUS*-ALS astrocytes activate the WNT/β-catenin pathway in *FUS*-ALS motor neurons

**Fig. 7:**
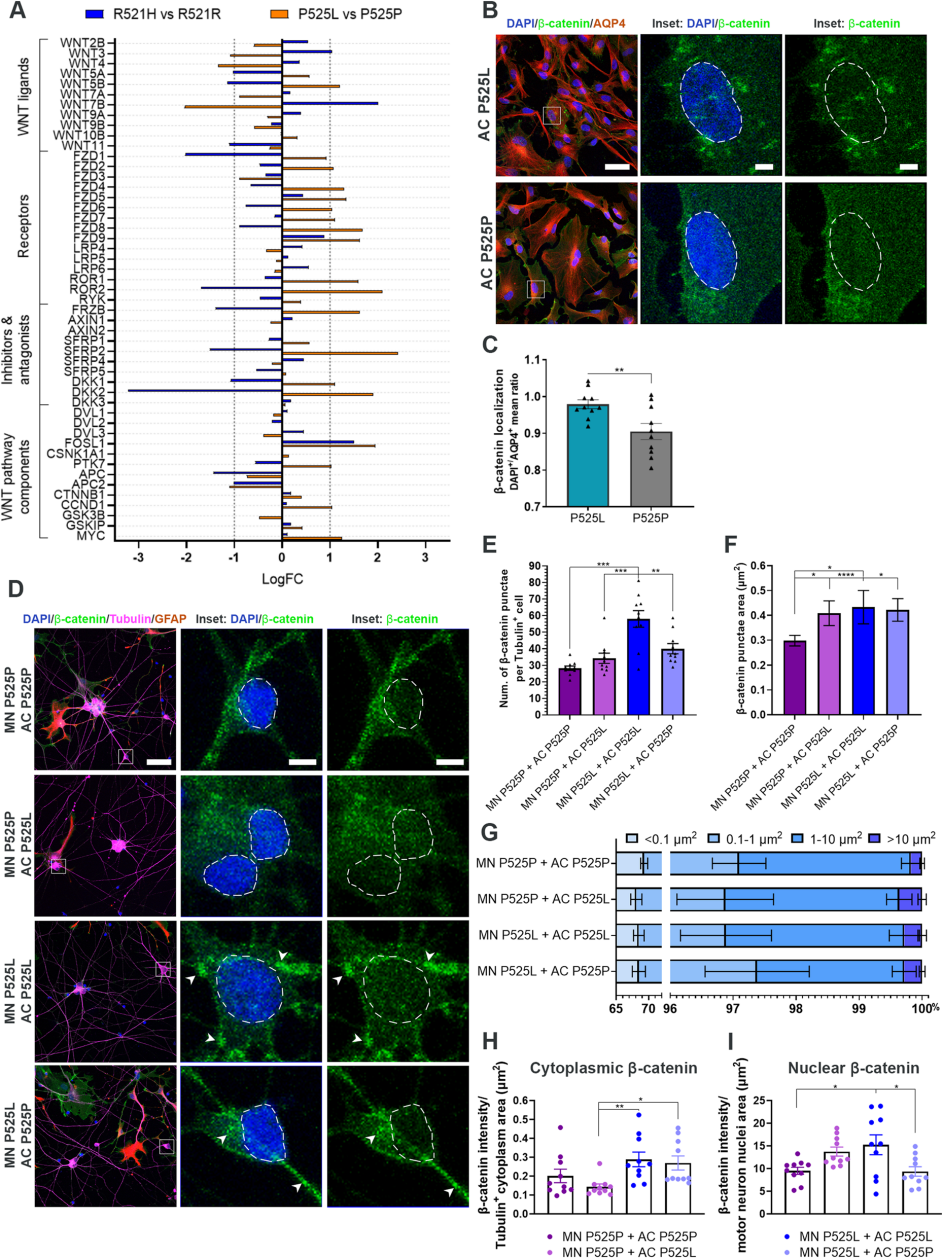
*FUS*-ALS astrocytes influence motor neurons through the WNT/β-catenin pathway. **A** RNAseq differential gene expression of WNT/β-catenin pathway components in 4-week mature astrocytes. **B** Representative confocal images of β-catenin expression in 4-week mature P525L and P525P astrocytes (AQP4). Inset shows a magnification of astrocytes with β-catenin localisation. Nuclei stained with DAPI. Scale bar: 50 μm. Inset scale bar: 5 μm. **C** Quantification of β-catenin localisation presented as the nuclei/cytoplasmic ratio. Data from 3 biological replicates (*n* = 30 images). Unpaired t test. **p < 0.01. **D** Representative confocal images of β-catenin expression in motor neurons (Tubulin), which have been cocultured with astrocytes (GFAP) for 48 h. Inset shows a magnification of motor neurons with β-catenin localisation. Arrowheads show examples of β-catenin accumulation. Nuclei (DAPI) are circled with a white dashed line. Scale bar: 50 μm. Inset scale bar: 5 μm. **E** Quantification of number of β-catenin accumulations per Tubulin^+^ motor neuron. **F** Quantification of individual β-catenin accumulation size in Tubulin^+^ motor neuron. **G** Percentage distribution of different size ranges of β-catenin accumulation in Tubulin^+^ motor neuron. **H** Quantification of cytoplasmic β-catenin expression per Tubulin^+^ motor neuron cytoplasmic area (μm^2^). **I** Quantification of nuclear β-catenin expression per Tubulin^+^ motor neuron nuclear area (μm.^2^). Panel (**E**-**I)** show mean ± s.e.m. of 3 biological replicates (*n* = 30 images). Panel (**E** and **I)**: One-way ANOVA with Tukey’s multiple comparisons test. Panel (**F** and **H**): Kruskal–Wallis test with Dunn’s multiple comparison test. *p < 0.05, **p < 0.01, ***p < 0.001 and ****p < 0.0001

Based on our RNAseq canonical pathway analysis (Fig. 4D), we identified one pathway, the WNT/β-catenin pathway, which was upregulated in both R521H and P525L astrocytes. Previous studies suggest the involvement of the WNT/β-catenin pathway in ALS [53, 54] as well as other neurodegenerative disorders [55], so to further investigate this, we looked into the gene expression of an array of WNT/β-catenin pathway components. Our data revealed a complex relationship of genes being both up- and downregulated (Fig. 7A). Target genes such as *CCND1*, *FZD4*, *FZD7*, *FZD8* and *MYC* were upregulated in P525L astrocytes, which correlated with an upregulation described in the final disease stages in *SOD1*-ALS mice [56]. Similarly, *FOSL1*, another important gene in late ALS disease stages [56], was upregulated in both R521H and P525L astrocytes. The activation of the WNT/β-catenin pathway causes translocation of β-catenin from the cytoplasm to the nucleus [57]. To investigate this further, we measured the nucleus/cytoplasmic ratio of β-catenin intensity in astrocytes, and found an increase in nuclear β-catenin in P525L astrocytes confirming an activation of the WNT/β-catenin pathway (Fig. 7B-C). To evaluate the influence of astrocytes on motor neurons, we cocultured P525L and P525P motor neurons and astrocytes for 48 h in 24-well plates, and evaluated the cellular localisation of β-catenin within Tubulin-positive motor neurons with ICC (Fig. 7D). At first, we observed a predominant accumulation of cytoplasmic β-catenin in P525L motor neurons cocultured with P525L astrocytes, which was successfully lowered by the presence of P525P astrocytes (Fig. 7E). Larger accumulations were found in P525P and P525L motor neurons cocultured with P525L astrocytes, while a smaller β-catenin cluster size was found in P525L motor neurons cultured with P525P astrocytes (Fig. 7F-G). Overall, the cytoplasmic β-catenin expression was less pronounced in the motor neurons in the fully isogenic cocultures and in the combined cocultures of P525P motor neurons and P525L astrocytes (Fig. 7H). Remarkably, we found a higher level of nuclear-translocated β-catenin in the fully mutant cocultures, which could indicate that P525L astrocytes are able to activate the WNT/β-catenin pathway in the mutant motor neurons after as little as two days coculture (Fig. 7I). A similar trend was observed in the P525P motor neurons cocultured with P525L astrocytes, while fully isogenic cocultures as well as combined P525L motor neurons and P525P astrocytes showed low nuclear β-catenin expression. In conclusion, these results establish an important role of WNT/β-catenin pathway activation in *FUS*-ALS motor neuron-astrocyte crosstalk.

## Discussion

In this study, we investigated the influence of hiPSC-derived astrocytes on human motor units cultured in microfluidic devices in the context of *FUS*-ALS. We discovered that *FUS*-ALS astrocytes displayed increased reactivity through calcium transient hyperactivity, increased GFAP expression, cytoplasmic FUS mislocalisation and spontaneous secretion of inflammatory cytokines in comparison to isogenic control astrocytes. Once cocultured with hiPSC-derived motor neurons, we observed a *FUS*-ALS astrocyte-mediated lack of neurite network integration and a cytotoxic effect on motor neuron neurite outgrowth in addition to NMJ formation and functionality. IC astrocytes were able to either improve or fully rescue all these aberrations. Finally, our data argue for a synergistic loss-of-support/gain-of-toxicity astrocyte functionality and an important role of WNT/β-catenin pathway activation as molecular mechanisms in *FUS*-ALS.

Although astrocyte reactivity has been proposed to be induced by microglia [52], the lack of microglia in our study suggests otherwise. Even though microglia are the primary immune-reactive cell type in the brain, our data demonstrate that astrocytes are equally relevant to produce pro-inflammatory cytokines and thereby play a central role in the abnormal immune response documented in ALS [58]. The early secretion of immune-regulatory cytokines IL-4 and especially IL-10 by immature astrocytes correlates with an initial anti-inflammatory and neuroprotective phase in ALS, where astrocytes and microglia try to modulate the abnormal response [59]. Subsequently, this astrocyte secretion of IL-4 and IL-10 in addition to the downregulation of associated molecular functions in R521H astrocytes suggest an auto-regulatory attempt to counteract the pro-inflammatory reaction and cytotoxic consequences. In ALS, the neuroprotective phase is later taken over by a cytotoxic phase, which is mediated by neurotoxic microglia [59], but likewise correlates with our astrocyte secretome. Notably, the secretion of IL-6 and IL-8 appeared prominent, and especially secreted IL-8 has been shown to be increased in cerebrospinal fluid of sALS patients emphasizing a common nominator in ALS [60, 61]. Interestingly, we observed a differentiation-dependent increase in the secretion of cytokines. This could be related to the clinical severity of the different mutations, as the pro-inflammatory cytokine response was more pronounced early in immature P525L astrocytes and late in more mature R521H astrocytes. Similarly, we saw an increase in active astrocytes and GFAP expression, a more pronounced cytoplasmic mislocalisation of FUS protein, and a more prominent reactive gene expression in P525L astrocytes in line with a more aggressive astrocytic phenotype. Furthermore, we observed a larger cytotoxic effect of P525L astrocytes on motor neuron neurite outgrowth and NMJ formation. Collectively these data demonstrate how the juvenile-onset P525L mutation causes more aggressive phenotypes *in-vitro*. However, to fully establish whether our observations are truly due to a mutation-dependent effect, further studies incorporating additional hiPSC lines are needed.

Jointly, our data argue for a complex role of astrocytes in *FUS*-ALS. The reactivity, secretion of cytokines, impairment of motor neuron neurites and NMJs all support an astrocytic involvement through a gain-of-toxicity function. Although we do not observe any cell death in our cocultures, these findings complement previous studies in which ALS astrocytic cytotoxic phenotypes were linked to excitotoxicity and/or motor neuron death [9, 21, 22, 62–67]. The lack of cell death in our system could be due to technical differences between protocols and the use of favourable culture conditions rather than a specific ALS-related mutation effect. Interestingly, the IPA canonical pathway analysis revealed a dominant downregulation of pathways, which overall can be divided into their involvement in either an immune response or homeostatic maintenance of neuronal networks. For the former group, a downregulation of HOTAIR (HOX transcript antisense RNA), STAT3 (signal transducer and activator of transcription 3) and Interferon signalling among others suggests a collective attempt to inhibit an immune response. Especially, HOTAIR regulates the NFκB-pathway [68], which is a major activator of the immune system response [69] and is involved in several neurodegenerative disorders [29, 70] including ALS [71, 72]. Notably, PI3K/AKT and BEX2 signalling, which were upregulated in R521H and P525L astrocytes, respectively, are also involved in NFκB-activation [73–77], and this dysregulation supports the heterogeneous reactive response observed in our astrocytes. In addition, modulations of the NFκB-pathway in astrocytes have proven unsuccessful in slowing down disease progression, suggesting that the astrocytic involvement in ALS extends beyond this single pathway [72, 78]. For the latter group, axonal guidance, synaptogenesis, endocannabinoid-, CREB- and CDK5 signalling are all important for neurodevelopment, network modulation and homeostatic maintenance [79–81]. Downregulation of these pathways, in addition to downregulation of several important receptors (neurotransmitter, glutamate, acetylcholine, etc.) from our GO molecular function analysis, indicates a loss-of-support function in *FUS*-ALS astrocytes, and complements previous astrocyte-mediated loss-of-support mechanisms in ALS [15–19]. In favor of this conclusion, a recent meta-analysis on sequencing data from both hiPSC and mouse ALS astrocytes with various ALS mutations confirms this synergistic interplay between loss-of-support and gain-of-toxicity mechanisms within the gene-expression, and further establishes it as a general mechanism in ALS [82]. In this context, our comparative differential gene expression analysis also revealed some overlap in the transcriptomic profile between *FUS*-P525L, *SOD1* and *C9orf72* hiPSC-derived astrocytes [42, 49]. This suggest a potential commonality across multiple fALS mutations, and further studies might reveal similar cytotoxic phenotypes.

Finally, our data argue for the involvement of the WNT/β-catenin pathway as an important player in the molecular mechanism of *FUS*-ALS. The WNT/β-catenin pathway is widely involved in cell survival, axonal guidance and NMJ formation [57]. For example, WNT ligands WNT3, WNT4 and WNT11 are involved in NMJ formation specifically enhancing the clustering of AChR on muscle cells as well as motor neuron axon outgrowth during NMJ innervation [83, 84]. Similarly, dysregulation of WNT ligands causes reduced AChR clustering and consequently disassembly of NMJs [85–88], which emphasizes the importance of WNT/β-catenin homeostasis. As such, the abnormal WNT/β-catenin pathway activation in our *FUS*-ALS astrocytes could be a central underlying mechanism of NMJ pathology in our multicellular system. Similarly, abnormal astrocyte activation of WNT ligands can activate a pro-inflammatory response within the astrocytes themselves [89], which could explain our secretory profile. In line with our observations, increased cytoplasmic β-catenin in astrocytes, neuronal β-catenin accumulation and activation [90, 91], β-catenin nuclear translocation [92], and dysregulation of WNT/β-catenin pathway components have been reported in the spinal cord, NMJs and muscles of ALS patients and in transgenic animal and in vitro models [53, 93–98], which discloses WNT/β-catenin pathway activation as a general underlying mechanism of ALS. Interestingly, it was demonstrated that WNT/β-catenin pathway activation in astrocytes increased neuroprotection in response to oxidative stress and inflammation [99], subsequently sparking the hypothesis that the activation of the WNT/β-catenin pathway in astrocytes could be a neuroprotective attempt to counteract motor neuron pathology in ALS through activation of the pathway within motor neurons themselves [57]. In support of this neuroprotective hypothesis, our P525L astrocytes successfully activated the WNT/β-catenin pathway within P525L motor neurons. As such, the combined effect of anti-inflammatory cytokine secretion and WNT/β-catenin pathway activation might thereby be an attempt to counteract the motor neuron cytotoxicity in the system, raising the question if mutant astrocytes are “all bad”. The inability to rescue or minimize the cytotoxicity is likely due to the overwhelming opposing toxic effects of the mutant astrocyte reactivity equally fuelled by the astrocytic WNT/β-catenin pathway dysregulation. Since WNT/β-catenin pathway balance is important to maintain optimal cellular homeostasis and NMJ integrity, the trend in activation of the WNT/β-catenin pathway within P525P motor neurons likely contributed to the impairment in neurite outgrowth and NMJ formation inflicted by the P525L astrocytes.

Until recently, astrocyte heterogeneity was a collectively accepted term used for their morphological differences and spatial location within the central nervous system [50]. The dynamic interplay between loss-of-support and gain-of-toxicity functions within our astrocyte populations specifies a heterogeneous functionality and favours a larger yet-to-be explored role of astrocytes in ALS. Transplantation of healthy rat astrocytes into transgenic rats expressing mutant human SOD1 protein has been shown to reduce microgliosis, attenuate motor neuron loss and extend disease duration [100]. In line with this, our IC astrocytes were able to improve or rescue all aberrations, which suggests a beneficial effect of gene therapy targeting the dynamic and migratory astrocytes rather than the static and post-mitotic motor neurons. Moreover, our data on the involvement of astrocytes on NMJ pathology in an all-human system endorse previous findings, where hiPSC-derived astrocytes from sALS patients were injected into mice and caused NMJ denervation and subsequent motor deficits [11]. This similarity between *FUS*-ALS and sALS demonstrates how astrocyte-induced NMJ toxicity is a general mechanism in ALS. Further integration of additional cell types such as upper motor neurons, interneurons and other glial cells would advance our understanding of the disease process in addition to enhancing the value of our NMJ model in drug testing and therapy development.

## Conclusion

Our study demonstrates that astrocytes are important players in ALS pathogenesis causing impairment of motor neuronal network and NMJ formation and functionality through multiple gain-of-toxicity and loss-of-support mechanisms. Furthermore, we propose an astrocytic attempt to counteract the toxicity through WNT/β-catenin pathway upregulation in motor neurons, albeit with limited success. In addition, our fully human multicellular microfluidics model provides a platform for further studies and can be used for drug development and testing.

## Supplementary Information

**Additional file 1: Supplementary Table 1.** List of material and resources.

**Additional file 2: Supplemental Figure 1.** Astrocyte differentiation verification with bright-field microscopy, qPCR and RNAseq. Related to Fig. 1. **Supplemental Figure 2**. Astrocyte differentiation verification with ICC. Related to Fig. 1. **Supplemental Figure** **3.** Astrocyte reactivity analysis. Related to Figs. 1 and 2. **Supplemental Figure 4.** Astrocyte secretome analysis. Related to Fig. 2. **Supplemental Figure 5.** Motor neuron and myotube differentiation verification with ICC. Related to Fig. 3. **Supplemental Figure 6.** Astrocyte and motor neuron coculture and LDH analysis. Related to Fig. 4. **Supplemental Figure 7.** NMJ morphology. Related to Fig. 6. **Supplemental Figure 8.** Myotube innervation. Related to Fig. 6.

**Additional file 3. **Astrocyte calcium activity_P525P.

**Additional file 4. **Astrocyte calcium activity_P525L.

**Additional file 5. **Astrocyte calcium activity_R521R.

**Additional file 6. **Astrocyte calcium activity_R521H.

**Additional file 7. **NMJ functionality_MN_P525P_AC_P525P.

**Additional file 8. **NMJ functionality_MN_P525P_AC_P525L.

**Additional file 9. **NMJ functionality_MN_P525L_AC_P525L.

**Additional file 10. **NMJ functionality_MN_P525L_AC_P525P.

**Additional file 11. **NMJ functionality_MN_P525P.

**Additional file 12. **NMJ functionality_MN_P525L.

**Additional file 13. **NMJ functionality_Myotube positive control

## Data Availability

• RNAseq data have been deposited at GEO and are publicly available as of the date of publication. Accession numbers are listed in the Supplementary Table 1, Additional file 1. Microscopy data reported in this paper will be shared by the corresponding author upon reasonable request. • ImageJ and Nikon software scripts are available from the corresponding author upon reasonable request. • Any additional information required to reanalyse the data reported in this paper is available from the corresponding author upon reasonable request.

## Abbreviations

AC: Astrocyte
AChR: Acetylcholine receptor
ACTN2: α-Actinin
ADM: Astrocyte differentiation medium
ALDH1L1: Aldehyde Dehydrogenase 1 Family Member L1
ALS: Amyotrophic lateral sclerosis
AMM: Astrocyte maturation medium
APC: Astrocyte progenitor cell
AQP4: Aquaporin-4
BDNF: Brain-derived neurotrophic factor
BSA: Bovine serum albumin
Btx: α-Bungarotoxin
ChAT: Choline acetyltransferase
CNTF: Ciliary neurotrophic factor
EB: Embryoid body
EGF: Epidermal growth factor
fALS: Familial ALS
FBS: Fetal bovine serum
FDR: False discovery rate
FGF-2: Fibroblast growth factor 2
FTLD: Frontotemporal lobar degeneration
FUS: *FUS RNA binding protein*
GDNF: Glial cell line-derived neurotrophic factor
GFAP: Glial fibrillary acidic protein
GLM: Generalized linear model
GO: Gene ontology
hiPSC: Human induced pluripotent stem cell
HOTAIR: HOX transcript antisense RNA
IC: Isogenic control
ICC: Immunocytochemistry
IGF-1: Insulin-like growth factor
IPA: Ingenuity Pathway Analysis
KCl: Potassium chloride
LDH: Lactate dehydrogenase
MAP2: Microtubule-Associated Protein 2
MN: Motor neuron
MN-NPC: Motor neuron neural progenitor cell
MSD: Meso Scale Discovery
MyHC: Myosin heavy chain
MyoG: Myogenin
NDS: Normal donkey serum
NEFH: Neurofilament heavy chain
NIM: Neuronal induction medium
NMJ: Neuromuscular junction
NMM: Neuronal maturation medium
NPC: Neural progenitor cell
S100β: S100 calcium-binding protein β
sALS: Sporadic ALS
SEM: Scanning electron microscopy
SOX9: SRY-Box Transcription Factor 9
STAT3: Signal transducer and activator of transcription 3
SYP: Synaptophysin
TBST: Tris-buffered saline with Tween
TNF-α: Tumour necrosis factor α
Tubulin: βIII-tubulin
WB: Western blot
