# Supplementary material for: FUS-ALS hiPSC-derived astrocytes impair human motor units through both gain-of-toxicity and loss-of-support mechanisms
Source: Mol Neurodegener. 2023 Jan 18;18:5. doi: 10.1186/s13024-022-00591-3 (PMC9847053; doi:10.1186/s13024-022-00591-3)
Supplement: Supplementary file 1 — Additional file 1: Supplementary Table 1. List of material and resources. [file 13024_2022_591_MOESM1_ESM.docx]

**Supplementary table 1. List of material and resources**

| **REAGENT or RESOURCE** | **SOURCE** | **IDENTIFIER** |
| --- | --- | --- |
| **Antibodies** | | |
| Rabbit anti-S100β (dilution 1:1000) | Abcam | Cat#Ab52642;  RRID:AB_882426 |
| Mouse anti-aquaporin 4 (AQP4) (dilution 1:200) | Abcam | Cat#Ab9512;  RRID:AB_307299 |
| Mouse anti-ALDH1L1 (dilution 1:1000) | Abcam | Cat#Ab56777;  RRID:AB_940204 |
| Goat anti-SOX9 (dilution 1:400) | R&D systems | Cat#AF3075;  RRID:AB_2194160 |
| Rat anti-GFAP (dilution ICC 1:1000, WB 1:500) | Thermo Fisher Scientific | Cat#13-0300;  RRID:AB_2532994 |
| Rabbit anti-Microtubule-Associated Protein 2 (MAP2) (dilution 1:1000) | Millipore | Cat#Ab5622;  RRID:AB_91939 |
| Rabbit anti-neurofilament heavy chain (NEFH) (dilution 1:1000) | Abcam | CAT#AB8135; RRID:AB_306298 |
| Rabbit anti-synaptophysin (SYP) (dilution 1:1000) | Cell Signaling | Cat#5461S; RRID:AB_10698743 |
| Goat anti-choline acetyltransferase (ChAT) (dilution 1:500) | Millipore | Cat#ab144P; RRID:AB_2079751 |
| Rabbit anti-islet 1 (dilution 1:400) | Millipore | Cat#ab4326; RRID:AB_10563961 |
| Mouse anti-βIII-tubulin (dilution 1:500) | Abcam | Cat#ab7751; RRID:AB_306045 |
| Mouse anti-myosin heavy chain (MyHC) (dilution 1:20) | This paper | N/A |
| Mouse anti-titin (dilution 1:300) | Developmental Studies Hybridoma Bank | Cat#9D10; RRID:AB_528491 |
| Rabbit anti-myogenin (MyoG) (dilution 1:500) | Abcam | Cat#Ab124800; RRID:AB_10971849 |
| Rabbit anti-desmin (dilution 1:200) | Abcam | Cat#Ab15200; RRID:AB_301744 |
| Mouse anti-α-actinin (ACTN2) (dilution 1:500) | Sigma-Aldrich | Cat#A7811;  RRID:AB_476766 |
| Rabbit anti-FUS/TLS (dilution 1:200) | Proteintech | Cat#115701-AP; RRID:AB_2247082 |
| Rabbit anti-cleaved caspase 3 (dilution 1:400) | Cell Signaling | Cat#9661S;  RRID:AB_2341188 |
| α-bungarotoxin (Btx) Alexa Fluor^TM^ 555 (dilution 1:1000) | Thermo Fisher Scientific | Cat#B35451; RRID:AB_2617152 |
| Mouse anti-GAPDH (dilution WB 1:5000) | Santa Cruz | Cat#Sc-47724;  RRID:AB_627678 |
| Rabbit anti-β-catenin (dilution 1:100) | Cell Signaling | Cat#8480S;  RRID:AB_11127855 |
| **Chemicals, peptides, and recombinant proteins** | | |
| SB431542 | Tocris Bioscience | Cat#1614;  CAS: 301836-41-9 |
| LDN-193189 | Stemgent | Cat#04-0074-02;  CAS: 1062368-24-4 |
| Recombinant Murine fibroblast growth factor (FGF)-2 | Peprotech | Cat#450-33 |
| Recombinant Human Epidermal Growth Factor (EGF) | Prospec | Cat#CYT-217 |
| Recombinant Human Activin A | Thermo Fisher Scientific | Cat#PHC9564 |
| Recombinant Human insulin-like growth factor (IGF)-1 | Peprotech | Cat#100-11 |
| Recombinant Human Heregulinβ-1 | Peprotech | Cat#100-03 |
| Y-27632 | Merck Millipore | Cat#688001  CAS: 146986-50-7 |
| CHIR99021 | Tocris Biosciences | Cat#4423  CAS: 252917-06-9 |
| Retinoic acid | Sigma-Aldrich | Cat#R2625  CAS: 302-79-4 |
| Smoothened agonist | Merck Millipore | Cat#566660  CAS: 364590-63-6 |
| Recombinant Human Brain-derived neurotrophic factor (BDNF) | PeproTech | Cat#450-02 |
| Recombinant Human Glial cell line-derived neurotrophic factor (GDNF) | PeproTech | Cat#450-10 |
| DAPT | Tocris Biosciences | Cat#2634  CAS: 208255-80-5 |
| Recombinant Human Ciliary neurotrophic factor (CNTF) | PeproTech | Cat#450-13 |
| Ultroser G | Pall Corporation | Cat#15950-017 |
| Recombinant Human EGF | Peprotech | Cat#AF-100-15 |
| Insulin solution human | Sigma-Aldrich | Cat#I9278  CAS: 11061-68-0 |
| Recombinant human agrin protein | R&D Systems | Cat#6624-AG-050 |
| Laminin from Engelbreth-Holm-Swarm murine sarcoma basement membrane | Sigma-Aldrich | Cat#L2020-1MG  CAS: 114956-81-9 |
| Fluo-4 AM live cell dye (5μM) | Thermo Fisher Scientific | Cat#F14201; CAS: 273221-67-3 |
| **Critical commercial assays** | | |
| CyQUANT^TM^ LDH Cytotoxicity Assay Kit | Thermo Fisher Scientific | Cat#C20300 |
| V-Plex Plus Proinflammatory Panel 1 (human) kit | Meso Scale Diagnostics | Cat#K15049G-1 |
| **Deposited data** | | |
| RNA-sequencing data | This paper | GEO: GSE196219 |
| Human reference genome GRCh38 | Genome Reference Consortium | <https://www.ncbi.nlm.nih.gov/grc/human> |
| RNA-sequencing data | (Taha *et al.*, 2022) | GEO: GSE142730 |
| RNA-sequencing data | (Birger *et al.*, 2019) | Array Express: E-MTAB-10916 |
| **Experimental models: Cell lines** | | |
| *FUS*-mutant hiPSCs (*FUS*^R521H^, *FUS*^P525L^) | (Guo *et al.*, 2017; Wang *et al.*, 2018) | N/A |
| Isogenic control hiPSCs (*FUS*^R521R^, *FUS*^P525P^) | CellSystems | N/A |
| Healthy adult primary myoblasts | Laboratory of Lieven Thorrez (KULAK). This paper. | N/A |
| **Oligonucleotides** | | |
| GFAP Primer: Forward GTCCCCCACCTAGTTTGCAG | (Chandrasekaran *et al.*, 2021) | N/A |
| S100β Primer: Forward AGGCCGGTGGAAGGATTATG | (Chandrasekaran *et al.*, 2021) | N/A |
| AQP4 Primer: Forward ATTTGCAATCAATTATACTGGTGCC | (Chandrasekaran *et al.*, 2021) | N/A |
| Human reference Taqman primer ACTB. Primer 1: ACAGAGCCTCGCCTTTG. Primer 2: CCTTGCACATGCCGGAG | Integrated DNA technologies | N/A |
| **Software and algorithms** | | |
| NIS-Elements AR 4.30.02/5.40 software | Nikon | N/A |
| LAS X | Leica | N/A |
| Ingenuity pathway analysis (IPA) | QIAGEN Inc. (Krämer *et al.*, 2014) | <https://www.qiagenbioinformatics.com/products/ingenuitypathway-analysis>;  RRID:SCR_008653 |
| Panther 16.0 | (Thomas *et al.*, 2003; Mi, Muruganujan and Thomas, 2013) | <http://www.pantherdb.org/>;  RRID:SCR_004869 |
| ImageJ 1.52p software | NIH | <https://imagej.nih.gov/ij/>; RRID:SCR_003070 |
| Ilastik 1.3.3post1 Pixel Classification software | (Berg *et al.*, 2019) | <https://www.ilastik.org/>;  RRID:SCR_015246 |
| Prism 9.2.0 | GraphPad | <https://www.graphpad.com/scientific-software/prism/>;  RRID:SCR_002798 |
| **Other** | | |
| Microfluidic devices | Xona^TM^ Microfluidics | Cat#XC150; Cat#SND75 |
| Nikon TIE A1R confocal microscope | Nikon | RRID:SCR_020318 |
| Leica SP8 DMI8 confocal microscope | Leica | N/A |
