# Supplementary material for: FUS-ALS hiPSC-derived astrocytes impair human motor units through both gain-of-toxicity and loss-of-support mechanisms
Source: Mol Neurodegener. 2023 Jan 18;18:5. doi: 10.1186/s13024-022-00591-3 (PMC9847053; doi:10.1186/s13024-022-00591-3)
Supplement: Supplementary file 2 — Additional file 2: Supplemental Figure 1. Astrocyte differentiation verification with bright-field microscopy, qPCR and RNAseq. Related to Fig. 1. Supplemental Figure 2. Astrocyte differentiation verification with ICC. Related to Fig. 1. Supplemental Figure 3. Astrocyte reactivity analysis. Related to Figs. 1 and 2. Supplemental Figure 4. Astrocyte secretome analysis. Related to Fig. 2. Supplemental Figure 5. Motor neuron and myotube differentiation verification with ICC. Related to Fig. 3. Supplemental Figure 6. Astrocyte and motor neuron coculture and LDH analysis. Related to Fig. 4. Supplemental Figure 7. NMJ morphology. Related to Fig. 6. Supplemental Figure 8. Myotube innervation. Related to Fig. 6. [file 13024_2022_591_MOESM2_ESM.docx]

**Additional File 2**

**Supplemental table of contents**

**Supplemental figure 1.** Astrocyte differentiation verification with bright-field microscopy, qPCR and RNAseq. Related to figure 1.

**Supplemental figure 2**. Astrocyte differentiation verification with ICC. Related to figure 1.

**Supplemental figure** **3.** Astrocyte reactivity analysis. Related to figure 1 and 2.

**Supplemental figure 4.** Astrocyte secretome analysis. Related to figure 2.

**Supplemental figure 5.** Motor neuron and myotube differentiation verification with ICC. Related to figure 3.

**Supplemental figure 6.** Astrocyte and motor neuron coculture and LDH analysis. Related to figure 4.

**Supplemental figure 7.** NMJ morphology. Related to figure 6.

**Supplemental figure 8.** Myotube innervation. Related to figure 6.

**
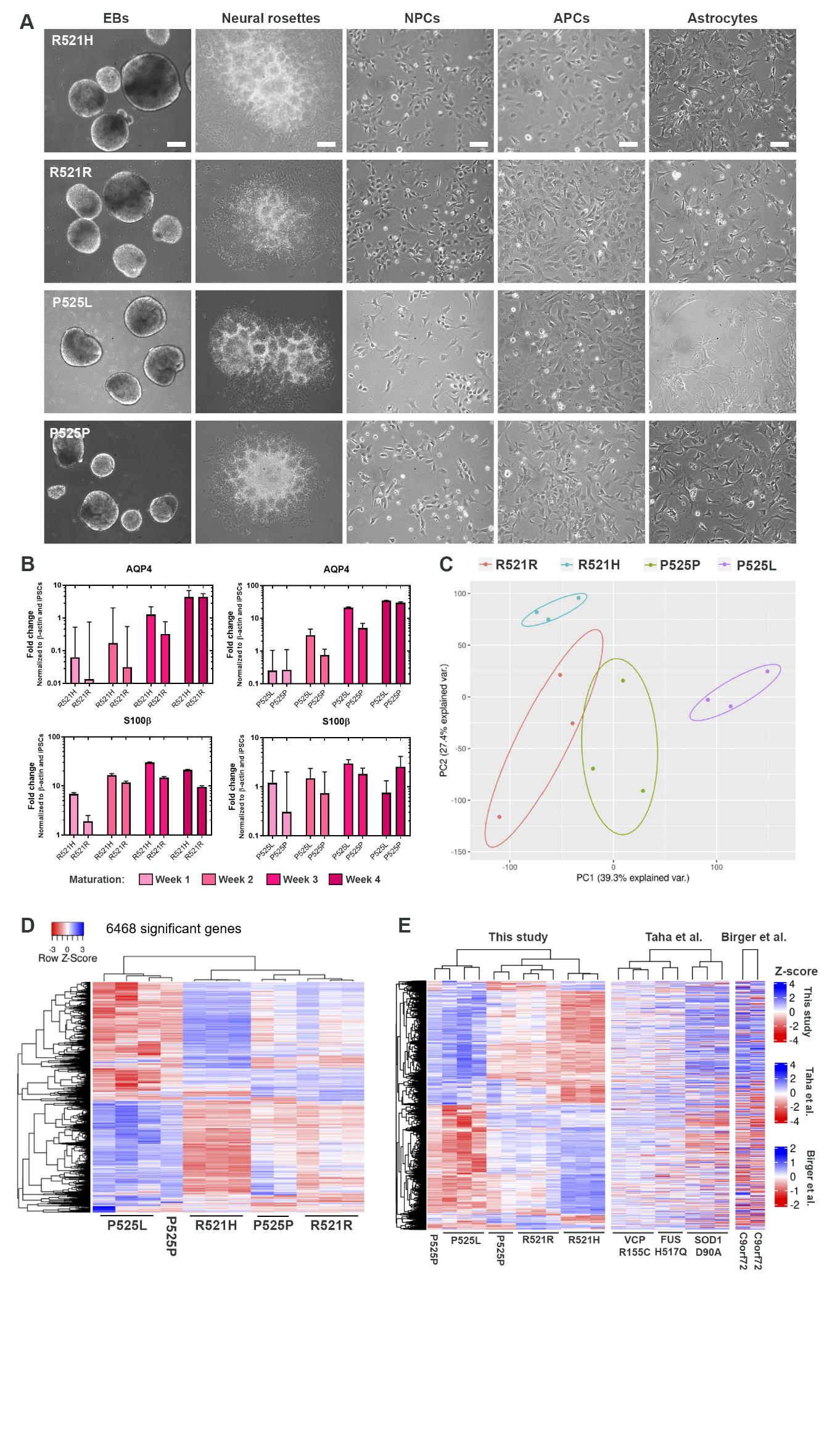
**

**Supplemental figure 1. Astrocyte differentiation verification with bright-field microscopy, qPCR and RNAseq**

**A.** Bright field images of astrocyte differentiation. EBs and Neural rosettes scale bar: 200 μm. NPCs, APCs and astrocytes scale bar: 100 μm. **B.** qPCR of AQP4 and S100β expression during week 1 to 4 of astrocyte maturation. Values normalized to β-actin and iPSCs. Mean + s.e.m. of 3 biological replicates with each 3 technical replicates. Y-axis = logarithmic scale. **C.** Bulk RNAseq principal component analysis of 3 biological replicates of astrocytes at 4 weeks of maturation. **D.** Heatmap and unsupervised hierarchical clustering of significant genes. Differentially expressed genes were selected based on FDR < 0.05. **E**. Heatmap and unsupervised hierarchical clustering of significant genes across multiple ALS mutations. Differentially expressed genes were selected based on FDR < 0.05. *SOD1*, *FUS* and *VCP* transcriptomic datasets are from Taha et al. (1) while *C9orf72* data are from Birger et al. (2).


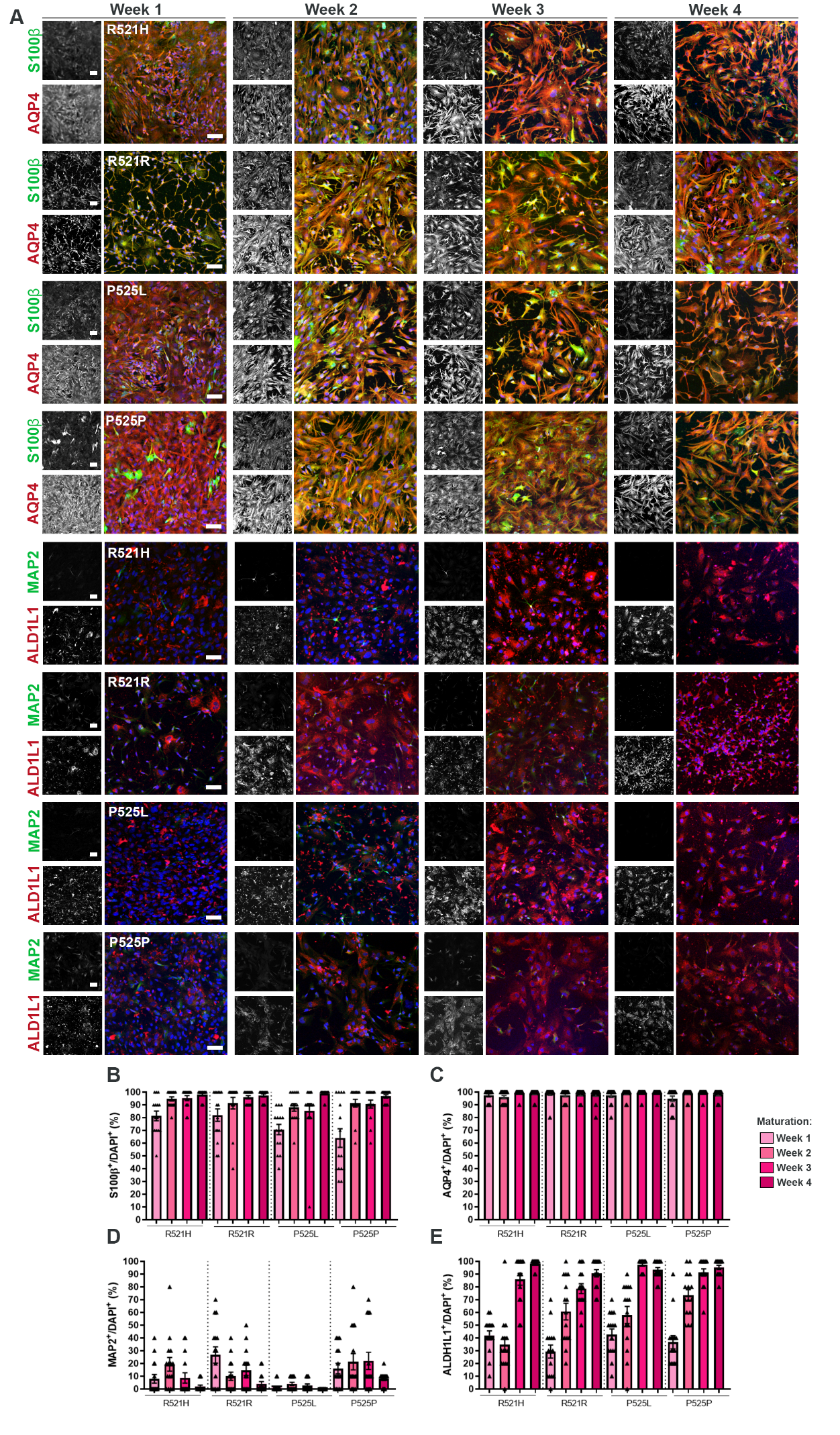


**Supplemental figure 2. Astrocyte differentiation verification with ICC**

**A.** Representative confocal images of hiPSC-derived astrocytes at week 1-4 of maturation stained with astrocyte specific markers S100β, AQP4, ALDH1L1 and neuronal marker MAP2. Nuclei stained with DAPI (blue). Scale bar: 75 μm. **B-E.** Quantification of the number of cells positive for S100β (panel **B**), AQP4 (panel **C**), MAP2 (panel **D**) and ALDH1L1 (panel **E**) during week 1-4 of maturation. Mean ± s.e.m. of 3 biological replicates (n=15 images). Kruskal-Wallis test with Dunn’s multiple comparisons test.


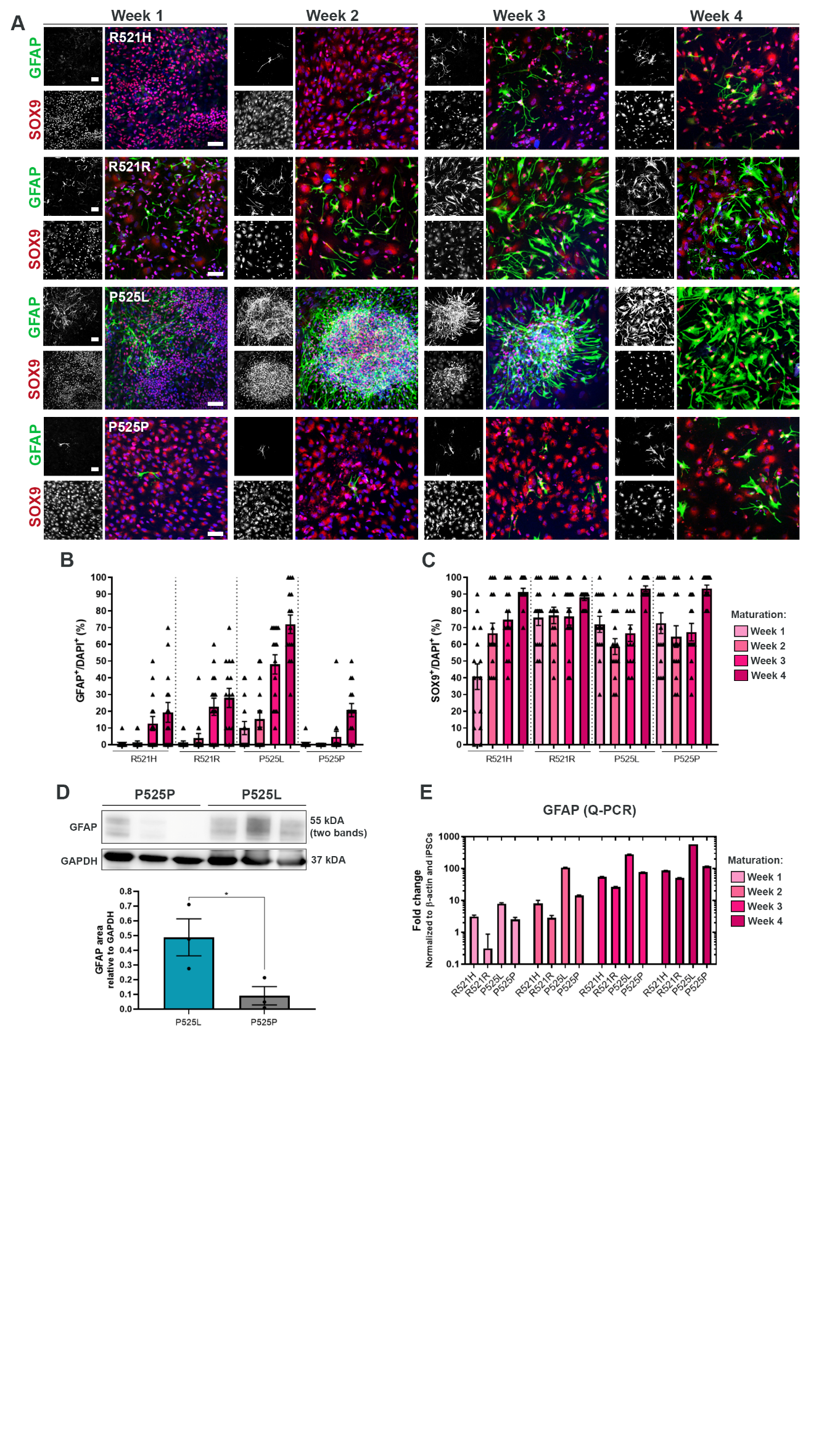


**Supplemental figure 3. Astrocyte reactivity analysis**

**A.** Representative confocal images of hiPSC-derived astrocytes at week 1-4 of maturation stained with markers GFAP and SOX9. Nuclei stained with DAPI. Scale bar: 75 μm. **B-C.** Quantification of the number of cells positive for GFAP (panel **B**) and SOX9 (panel **C**) during week 1-4 of maturation. Mean ± s.e.m. of 3 biological replicates (n=15 images). Kruskal-Wallis test with Dunn’s multiple comparisons test. **D.** Western blot with quantification of GFAP protein content relative to GAPDH in astrocytes at week 4 of maturation. Mean ± s.e.m. of 3 biological replicates. Unpaired t test. *p < 0.05. **E.** qPCR of GFAP expression during week 1 to 4 of astrocyte maturation. Values normalized to β-actin and iPSCs. Mean + s.e.m. of 3 biological replicates with each 3 technical replicates. Y-axis = logarithmic scale.


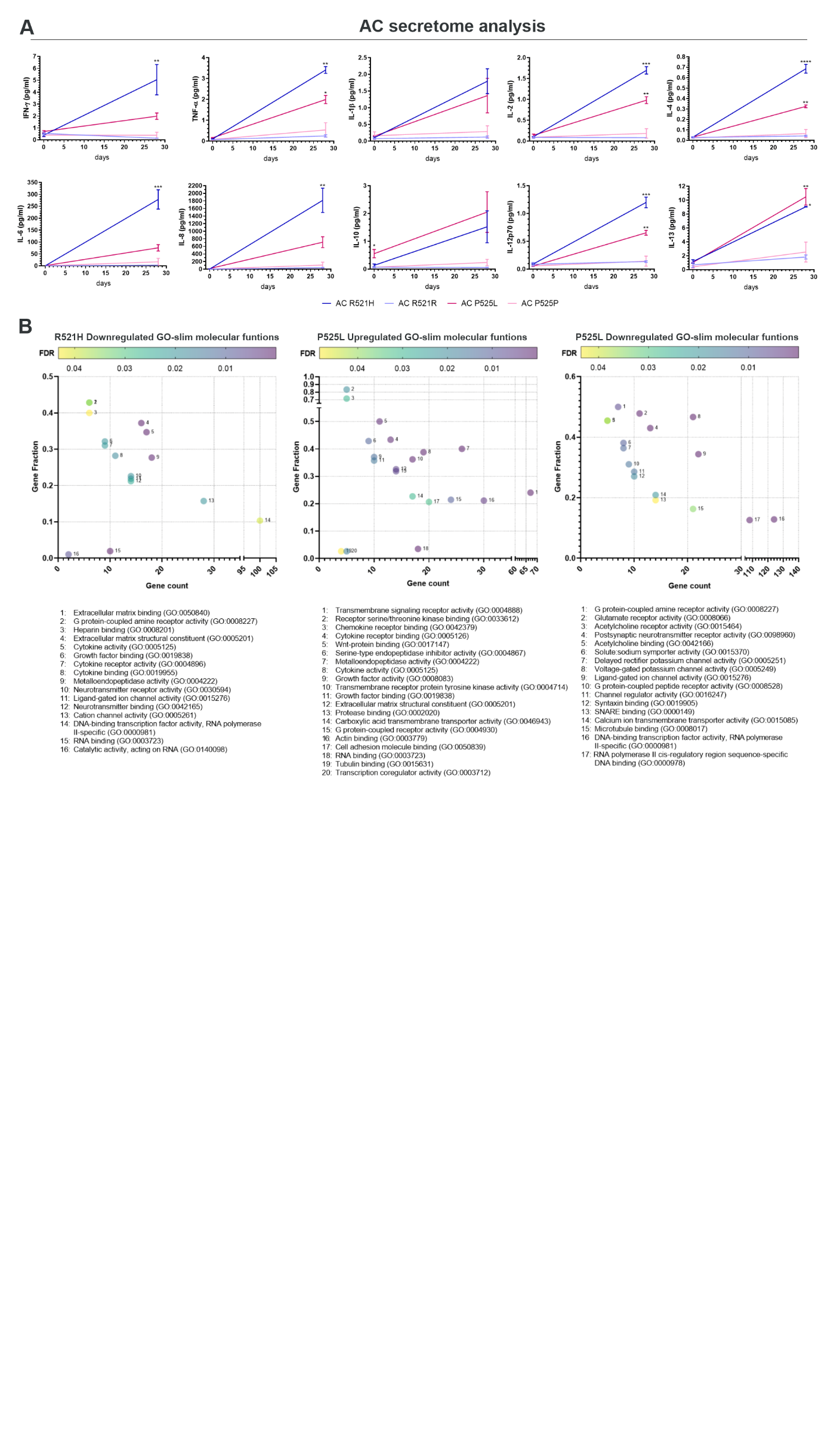


**Supplemental figure 4. Astrocyte secretome analysis**

**A.** Time course of pro-inflammatory cytokine secretion during astrocyte maturation from immature astrocytes (d0) to mature astrocytes (d28 = week 4). Mean ± s.e.m. of 3 biological replicates. One-way ANOVA with Tukey’s multiple comparisons test. *p < 0.05, **p < 0.01, ***p < 0.001 and ****p < 0.0001 **B.** Bulk RNAseq Panther Gene Ontology-slim molecular function analysis on up- and downregulated genes in week 4 mature astrocytes. Differentially expressed genes with log_2_FC < -1 were considered downregulated, while genes with log_2_FC > 1 were considered upregulated. False discover rate (FDR) < 0.05 was considered significant. Fisher’s exact test.


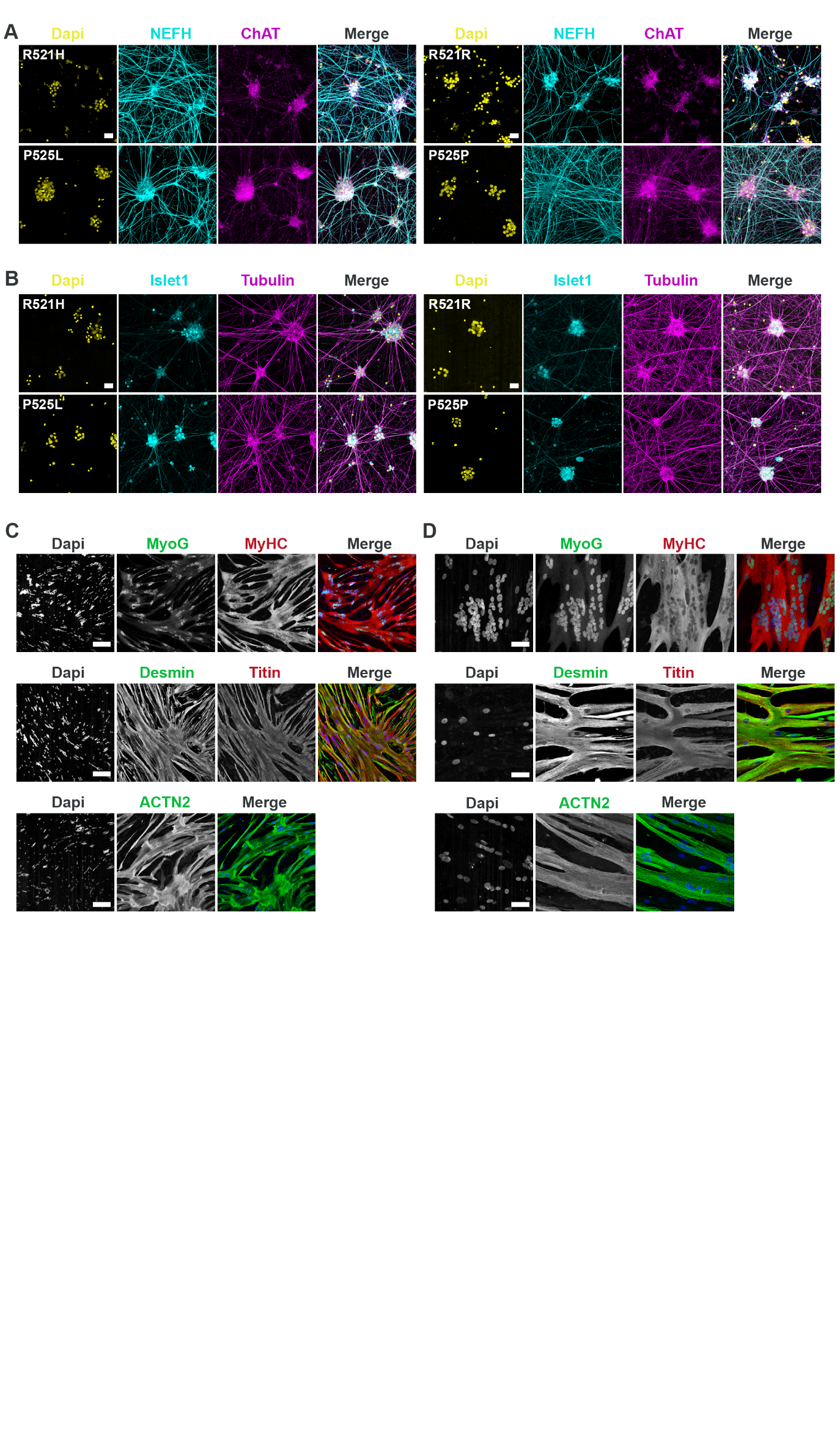


**Supplemental figure 5. Motor neuron and myotube differentiation verification with ICC**

**A-B.** Representative confocal images of mature motor neurons at day 28 of differentiation stained with markers **A**: NEFH and ChAT, **B**: Islet-1 and βIII-tubulin (Tubulin). Nuclei stained with DAPI. Scale bar: 25 μm. **C-D.** Representative confocal images of myotubes stained with MyoG, MyHC, desmin, titin and ACTN2. Nuclei stained with DAPI. Scale bar (panel **C**): 200 μm. Scale bar (panel **D**): 50 μm.


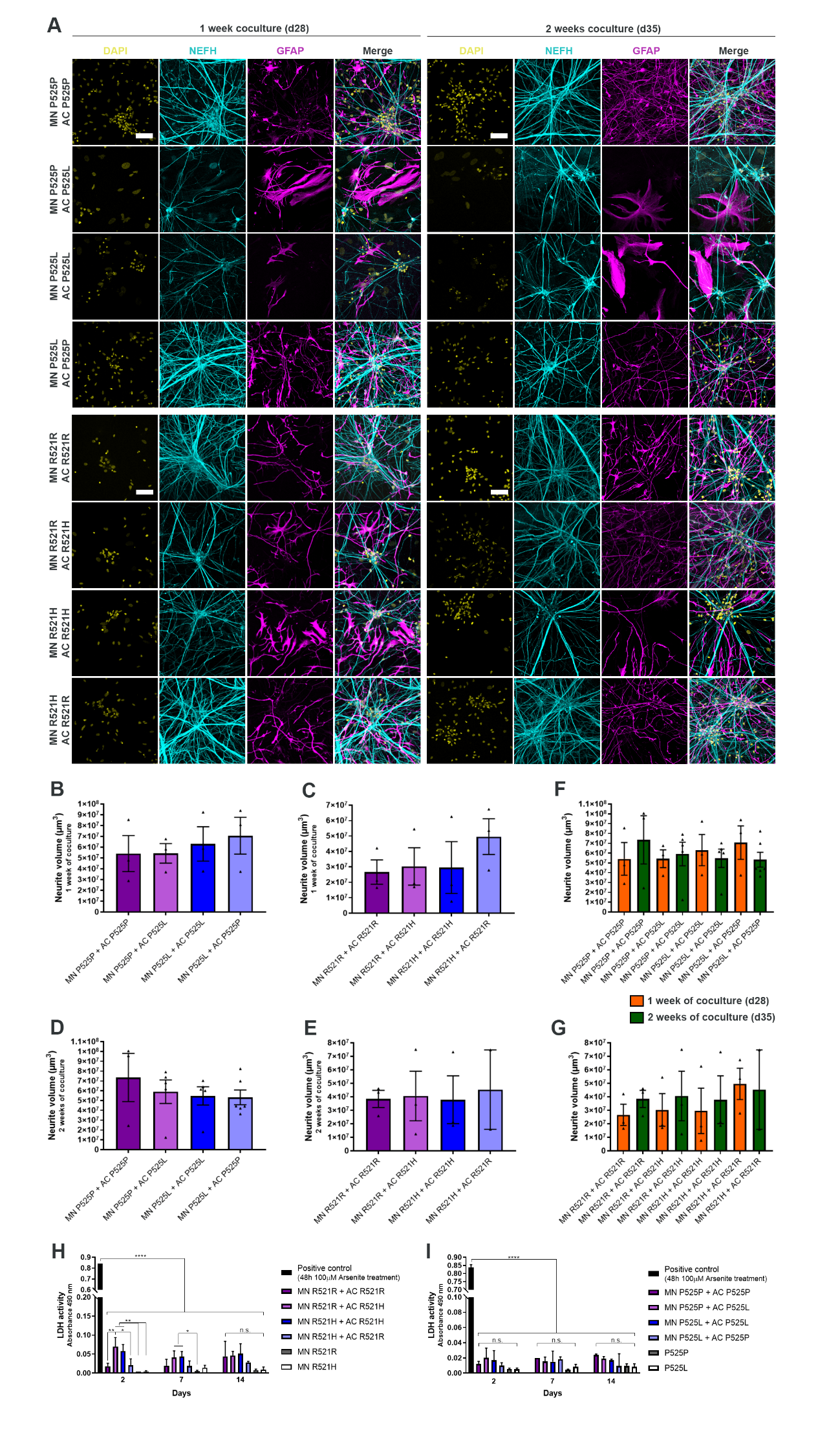


**Supplemental figure 6. Astrocyte and motor neuron coculture and LDH analysis**

**A.** Representative confocal images of motor neuron (NEFH) and astrocyte (GFAP) cocultures in microfluidic devices after 1 and 2 weeks. Nuclei stained with DAPI. Scale bar: 50 μm. **B-G**. Quantification of motor neuron neurite (NEFH) volume (μm^3^) in motor neuron/astrocyte compartment of microfluidic devices after 1(panel **B**-**C**) and 2 (panel **D**-**E**) weeks of coculture. Panel **F-G** shows volume over time. **H-I.** LDH activity measurement in media supernatant after 2, 7 and 14 days of coculture between motor neurons and astrocytes. Monocultured and 48h 100 μM arsenite-treated motor neurons were included as controls. Data in **B**-**G** represent mean ± s.e.m. from 3-4 biological replicates. Data in **H-I** represent mean ± s.e.m. of 3 biological replicates. One-way ANOVA with Tukey’s multiple comparisons test. *p < 0.05, **p < 0.01 and ****p < 0.0001.


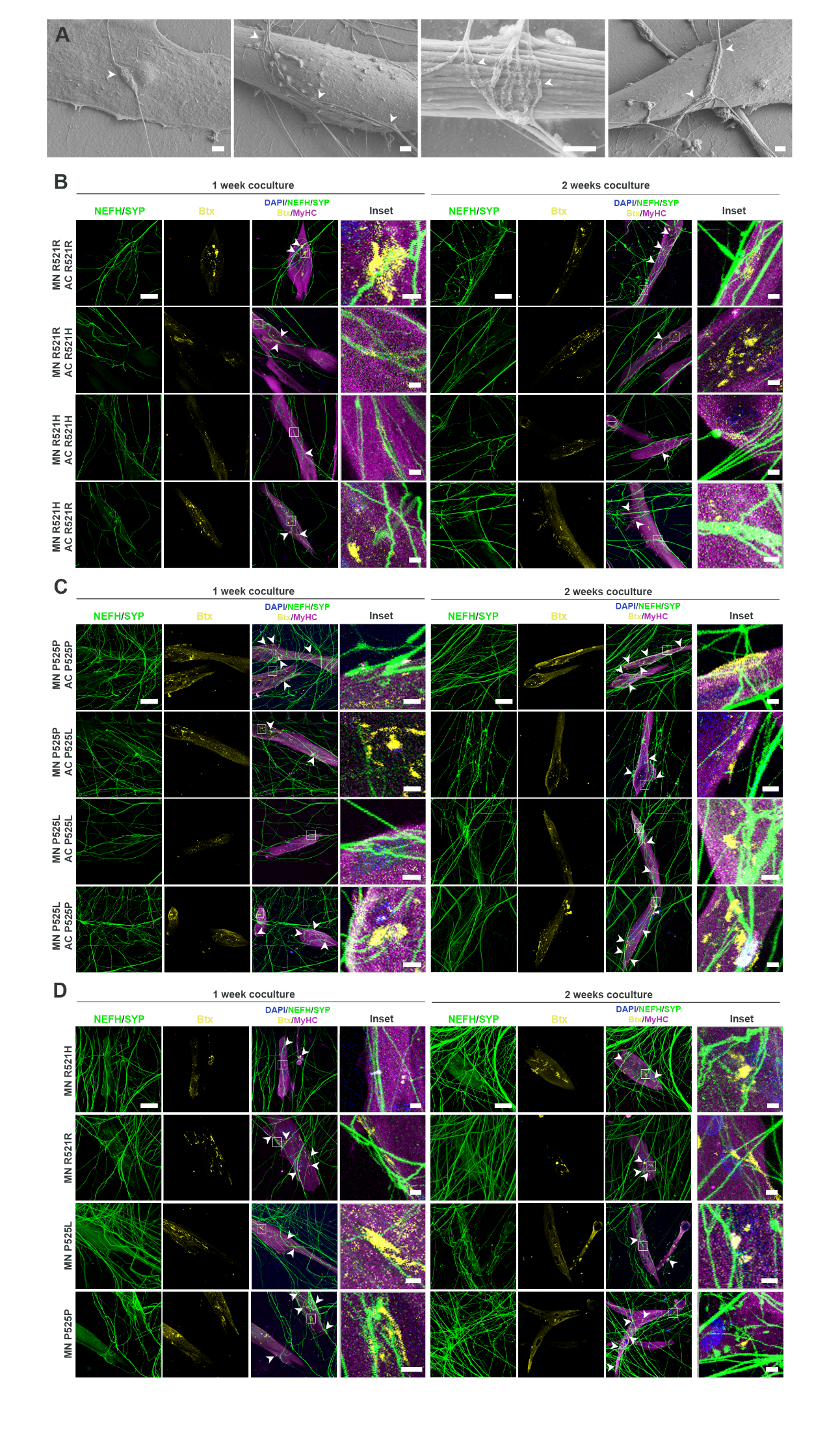


**Supplemental figure 7. NMJ morphology**

**A.** Scanning electron microscopy images of different NMJ morphology after 1 week of motor neuron/astrocyte cocultures according to the coculture timeline. Arrows mark NMJs. Scale bar: 2 μm. **B-D.** Representative confocal images of NMJ formation in R521R and R521H motor neuron/astrocyte cocultures (panel **B**), P525P and P525L motor neuron/astrocyte cocultures (panel **C**) and motor unit cultures without astrocytes (panel **D**) after 1 and 2 weeks according to the coculture timeline. NMJs were identified through colocalisation (arrow) between motor neuron (NEFH) and presynaptic (SYP) markers with postsynaptic AChR marker Btx on MyHC-stained myotubes. Scale bar: 50 μm. Inset scale bar: 5 μm.


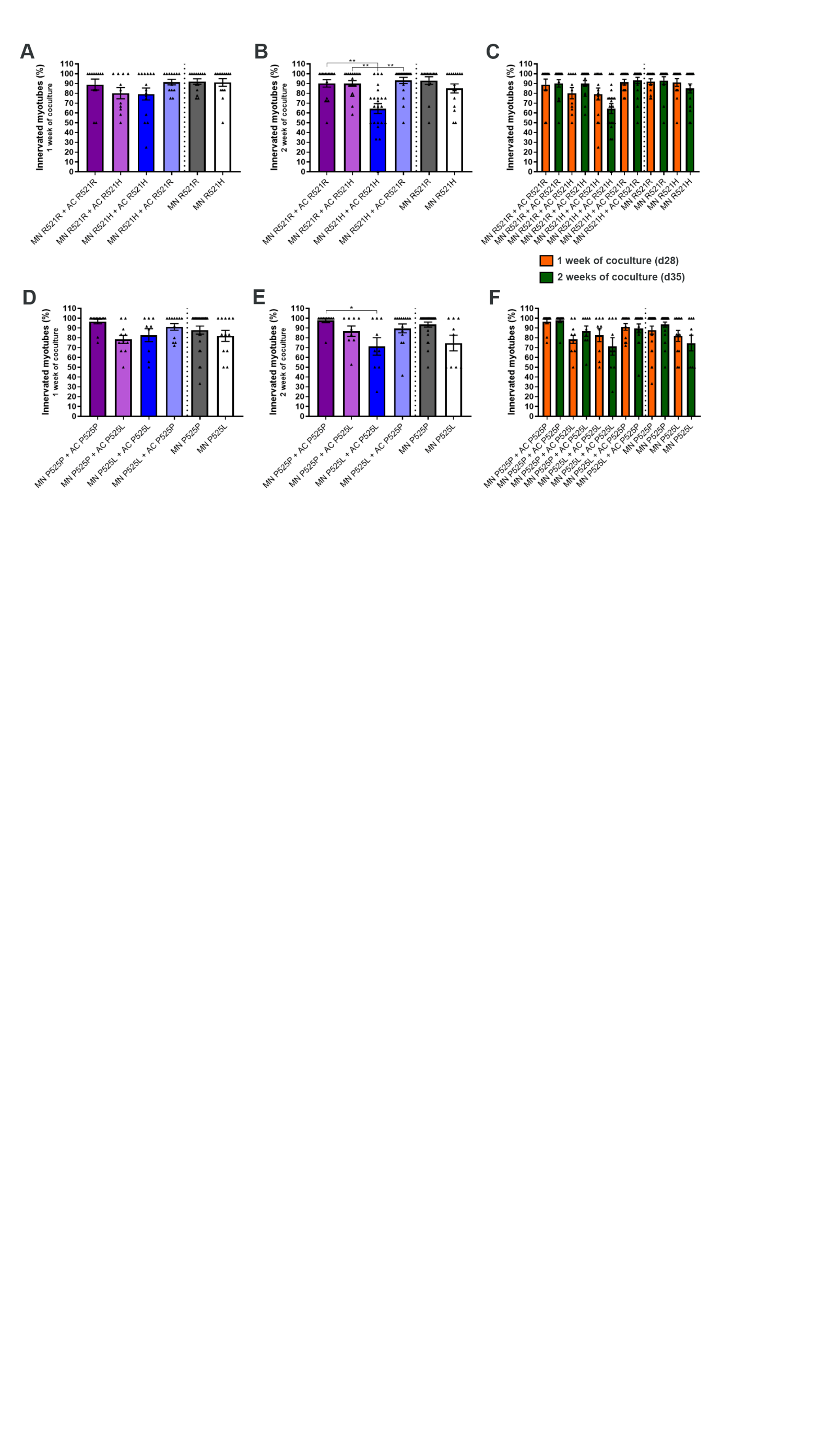


**Supplemental figure 8. Myotube innervation**

Quantifications of myotube innervation after 1 week (panel **A** and **D**) and 2 weeks (panel **B** and **E**) of cocultures between motor neuron/astrocytes and myotubes. Panel **C** and **F** show the percentage of innervated myotubes over time. Graphs depict mean ± s.e.m. of 3 biological replicates. One-way ANOVA with Tukey’s multiple comparisons test. *p < 0.05 and **p < 0.01.

**References:**

1. Taha DM, Clarke BE, Hall CE, Tyzack GE, Ziff OJ, Greensmith L, et al. Astrocytes display cell autonomous and diverse early reactive states in familial amyotrophic lateral sclerosis. Brain. 2022;145(2):481–9.

2. Birger A, Ben-Dor I, Ottolenghi M, Turetsky T, Gil Y, Sweetat S, et al. Human iPSC-derived astrocytes from ALS patients with mutated C9ORF72 show increased oxidative stress and neurotoxicity. EBioMedicine. 2019;50:274–89.
